# Supplementary material for: An allosteric inhibitor of RhoGAP class-IX myosins suppresses the metastatic features of cancer cells
Source: Nat Commun. 2024 Nov 16;15:9947. doi: 10.1038/s41467-024-54181-6 (PMC11569205; doi:10.1038/s41467-024-54181-6)
Supplement: Supplementary file 1 — Supplementary Information [file 41467_2024_54181_MOESM1_ESM.pdf]

An allosteric inhibitor of RhoGAP class-IX myosins suppresses the metastatic features of cancer cells

Despoina Kyriazi[1], Lea Voth[1], Almke Bader[1], Wiebke Ewert[1,2], Juliane Gerlach[3], Kerstin Elfrink[4], Peter Franz[1], Mariana I. Tsap[5], Bastian Schirmer[6], Julia Damiano-Guercio[1], Falk K. Hartmann[1], Masina Plenge[7], Azam Salari[8], Dennis Schoettelndreier[1], Katharina Strienke[1], Nadine Bresch[1], Claudio Salinas[1], Herwig O. Gutzeit[9], Nora Schaumann[10], Kais Hussein[11], Heike Bähre[12], Inga Brüsch[13], Peter Claus[14], Detlef Neumann[6], Manuel H. Taft[1], Halyna R. Shcherbata[5], Anaclet Ngezahayo[7], Martin Bähler[4], Mahdi Amiri[8], Hans-Joachim Knölker[3], Matthias Preller[1,2], and Georgios Tsiavaliaris[1,*]

[1]Institute for Biophysical Chemistry, Hannover Medical School, Hannover, Germany; [2]Institute for Functional Gene Analytics (IFGA), Bonn-Rhein-Sieg University of Applied Sciences, Rheinbach, Germany; [3]Faculty of Chemistry, TU Dresden, Dresden, Germany; [4]Institute of Integrative Cell Biology and Physiology, University of Münster, Münster, Germany; [5]Institute of Cell Biochemistry, Hannover Medical School, Hannover, Germany; [6]Institute of Pharmacology, Hannover Medical School, Hannover, Germany; [7]Department of Cell Physiology and Biophysics, Institute of Cell Biology and Biophysics, Leibniz Universität Hannover, Hannover, Germany; [8]Department of Gastroenterology, Hepatology and Endocrinology, Hannover Medical School, Hannover, Germany; [9]Department of Biology, TU Dresden, Dresden, Germany.[10]Institute for Pathology, Hannover Medical School, Hannover, Germany; [11]Institute of Pathology, KRH Klinikum Nordstadt, Hannover. [12] Research Core Unit Mass Spectrometry - Metabolomics, Hannover Medical School, Germany; [13]Institute for Laboratory Animal Science, Hannover Medical School, Hannover, Germany; [14]SMATHERIA gGmbH – Non-Profit Biomedical Research Institute, Hannover, Germany.

*Correspondence: Tsiavaliaris.Georgios@mh-hannover.de (G.T.)

SUPPLEMENTARY INFORMATION

- Supplementary Figures
- Supplementary Tables with legends
- Supplementary Methods
    - Reagent and resource information
    - Experimental procedures of compound syntheses
    - NMR Spectra
- Supplementary References

SUPPLEMENTARY FIGURES

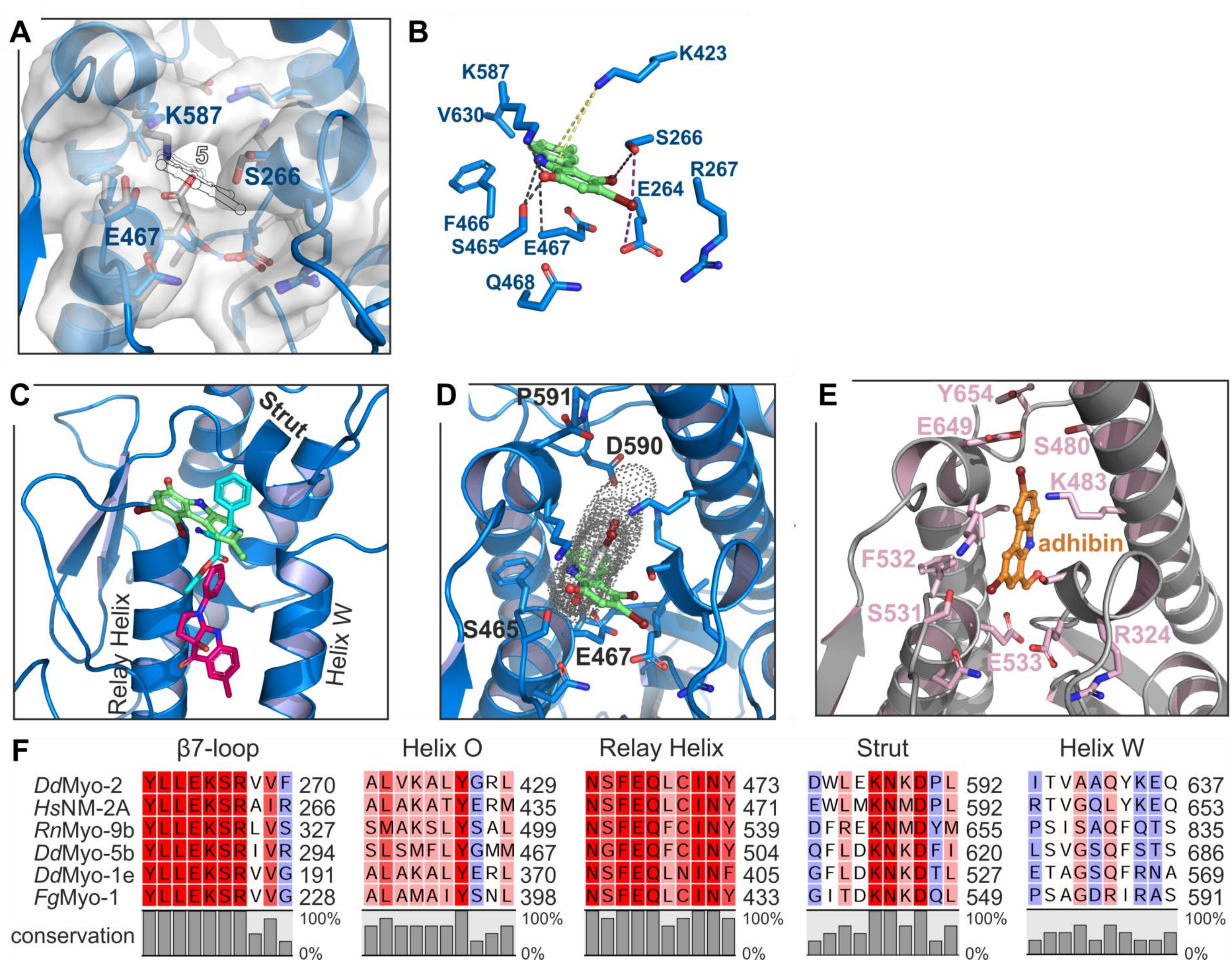

Supplementary Figure 1. Structural analysis of inhibitor binding site. **A** Sidechains of residues Glu467, Ser266, and Lys587 rearrange to form the allosteric pocket allowing binding of the adhibin analogue **5** (silhouette). **B** Residues in the binding pocket involved in drug coordination (dashed lines): hydrogen bonds are shown in grey, cation-π-interactions in yellow, halogen bonds in purple, and hydrophobic interactions. **C** Superposition of myosin crystal structures showing the binding geometry of **5** (green) and phenamacril (pdb: 6ui4; cyan) in the binding pocket. **D** Comparison of the binding pose of **5** in the crystal structure and after redocking. **E** *In silico* docking of adhibin (orange) to the homology model of the motor domain of *Rn*myosin-9b predicts a slightly different orientation of adhibin in the binding pocket interacting preferentially with amino acids E649 and Y654 part of the strut loop that connects the upper and lower domains forming the actin-binding cleft of myosin. **F** Sequence alignment of *Dd*myosin-2, -5b and -1E with *Rn*myosin-9b, *Hs*NM2A, and *Fg*myosin-1 reveals a high degree of conservation of the structural elements involved in the formation of the inhibitor binding site.

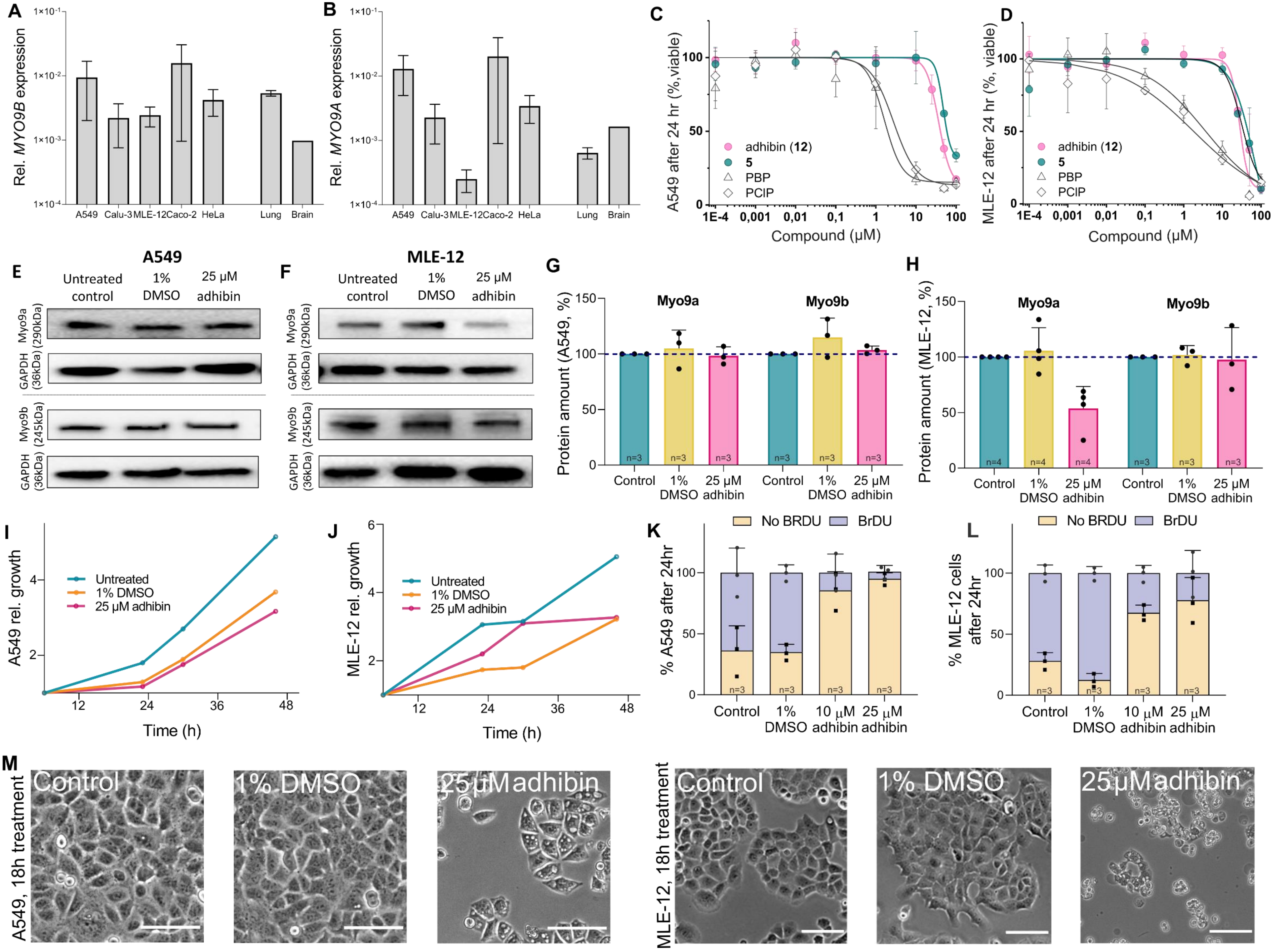
A
Rel. MYO9B expression
A549
Calu-3
MLE-12
Caco-2
HeLa
Lung
Brain
B
Rel. MYO9A expression
C
A549 after 24 hr (%, viable)
adhibin (12)
5
PBP
PCIP
Compound (µM)
D
MLE-12 after 24 hr (%, viable)
E
A549
Untreated control
1% DMSO
25 µM adhibin
Myo9a (290kDa)
GAPDH (36kDa)
Myo9b (245kDa)
F
MLE-12
G
Protein amount (A549, %)
Myo9a
Myo9b
Control
1% DMSO
25 µM adhibin
H
Protein amount (MLE-12, %)
I
A549 rel. growth
Untreated
1% DMSO
25 µM adhibin
Time (h)
J
MLE-12 rel. growth
K
No BRDU
BrDU
% A549 after 24hr
10 µM adhibin
L
% MLE-12 cells after 24hr
M
A549, 18h treatment
Control
1% DMSO
25µM adhibin
MLE-12, 18h treatment

Supplementary Figure 2. Adhibin impairs growth, adhesion, and proliferation of epithelial cancer cells. **A** *MYO9a* and **B** *MYO9b* expression in A549, Calu-3, MLE-12, CaCo-2, HeLa cells, lung and brain tissue. **C,D** Neutral red uptake assays showing viability of A549 and MLE-12 cells after 18 hour cultivation in medium supplemented with increasing concentrations of adhibin, the adhibin analogue **5**, PBP, and PClP. Error bars represent mean ± s.e.m from at least three independent experiments. **E,F** Western blots showing Myo9a and Myo9b protein content in lysates of A549 and MLE-12 cells in the absence and presence of adhibin relative to GAPDH. n is the number of experiments. Error bars represent mean ± S.D.. **G,H** Quantification of Myo9a and Myo9b protein content. n is the number of experiments. Error bars represent mean ± S.D.. **I,J** Growth of A549 and MLE-12 cells in medium containing 1% DMSO, 10 µM and 25 µM adhibin, respectively. **K,L** BrDU-based proliferation assays showing percentage of A549 and MLE-12 cells progressing through S-phase, 24 h after treatment. n is the number of experiments. Error bars represent mean ± S.D.. **M** Representative bright field images showing cell monolayers of A549 and MLE12 cells with and without adhibin treatment. Scale bars: 20 µm.

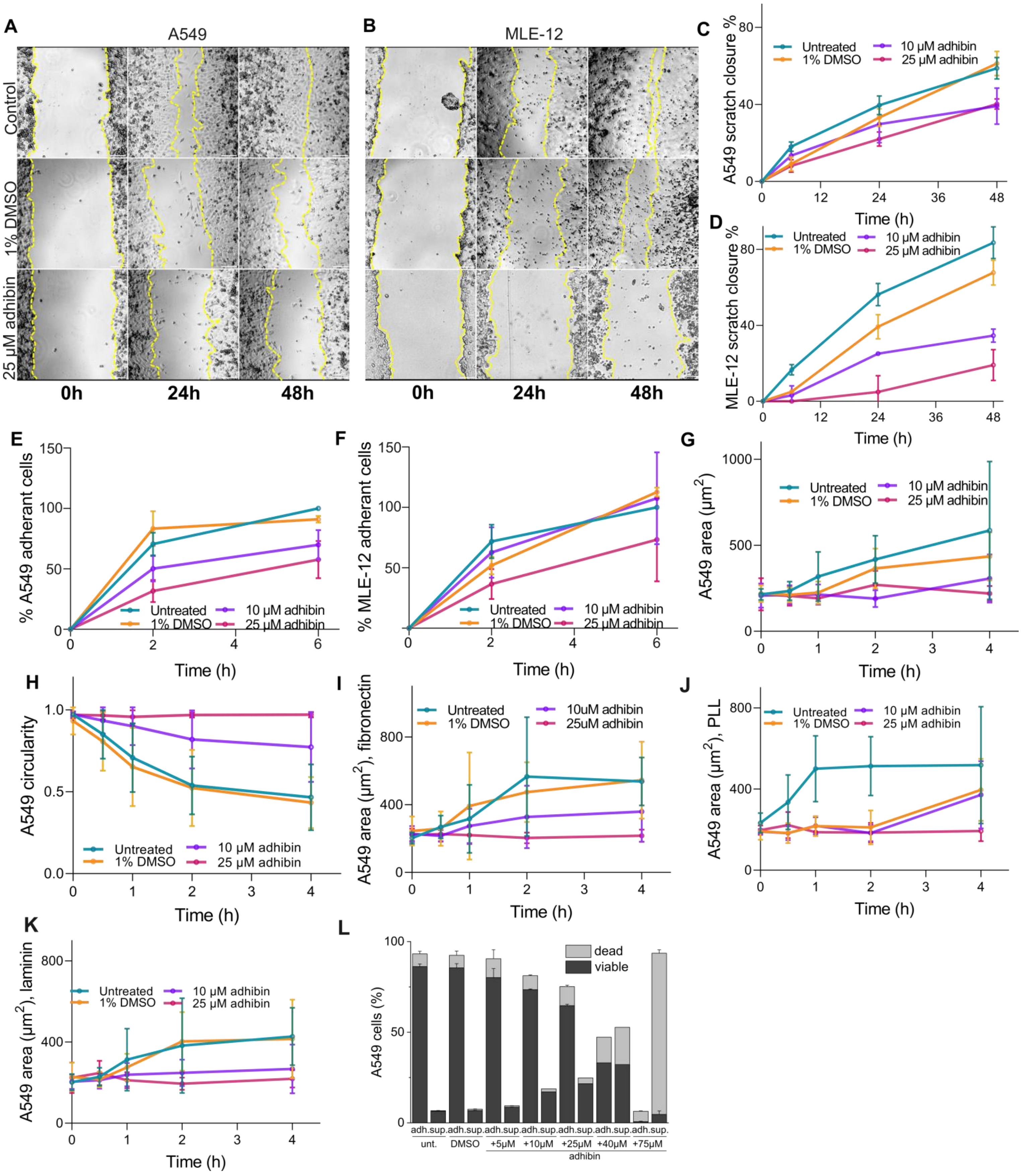
A
A549
B
MLE-12
Control
1% DMSO
25 μM adhibin
0h
24h
48h
C
A549 scratch closure %
Time (h)
D
MLE-12 scratch closure %
Untreated
1% DMSO
10 μM adhibin
25 μM adhibin
E
% A549 adherant cells
F
% MLE-12 adherant cells
G
A549 area (μm2)
H
A549 circularity
I
A549 area (μm2), fibronectin
10uM adhibin
25uM adhibin
J
A549 area (μm2), PLL
K
A549 area (μm2), laminin
L
A549 cells (%)
dead
viable
adh. sup.
unt.
DMSO
+5μM
+10μM
+25μM
+40μM
+75μM
adhibin

Supplementary Figure 3. Adhibin impairs surface adhesion and migration of epithelial cancer cells. A,B Representative wound healing assays showing scratch closure of control, 1% DMSO and 25µM adhibin treated A549 and MLE-12 cells at 0, 24, and 48 h of cultivation. Scale bars: 100 µm. **C,D** Progress of scratch closure of A549 and MLE-12 cells under the respective conditions. Mean ± s.e.m. from three independent experiments. **E,F** Percentage of surface adhered A549 and MLE-12 cells in the absence and presence of 10 and 25 µM adhibin at defined time points (0.5 to 6 h). Error bars represent mean ± s.e.m from three independent experiments. **G** Area of A549 cells upon seeding on uncoated glass after 10 min, 30 min, 1 h, 2 h and 4 h exposures to 10 and 25 µM adhibin in comparison to control conditions (untreated, 1% DMSO). Error bars represent mean ± S.D. from three independent experiments. **H** Circularity of A549 cells upon seeding on uncoated glass after 0 hr, 30 min, 1 hr, 2 hr and 4 hr exposures to 10 and 25 µM adhibin in comparison to control conditions (untreated, 1% DMSO). Error bars represent mean ± S.D. from three independent experiments. **I-K** Area of A549 cells upon seeding on PLL, fibronectin and laminin after 10 min, 30 min, 1 h, 2 h and 4 h exposures to 10 and 25 µM adhibin in comparison to control conditions (untreated, 1% DMSO). Error bars represent mean ± S.D. from three independent experiments. **L** Percentage of viable and dead cells in the absence and presence of increasing concentrations of adhibin after 6 h cultivation. Quantification was performed for both, surface adhered (pel.) and detached, floating (sup.) cells.

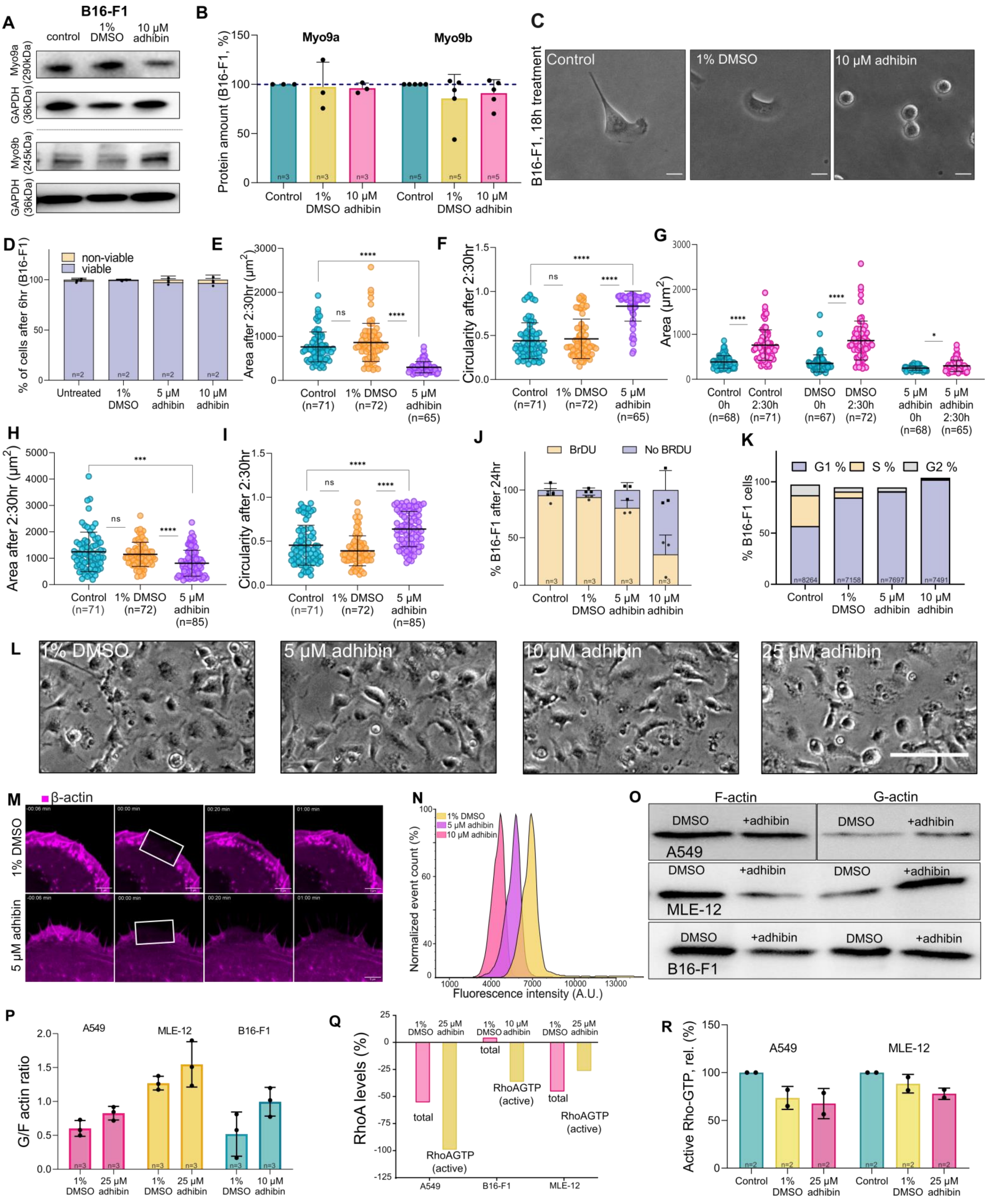

A
B16-F1
control
1% DMSO
10 µM adhibin
Myo9a (290kDa)
GAPDH (36kDa)
Myo9b (245kDa)
GAPDH (36kDa)
B
Myo9a
Myo9b
Protein amount (B16-F1, %)
Control
1% DMSO
10 µM adhibin
C
B16-F1, 18h treatment
Control
1% DMSO
10 µM adhibin
D
non-viable
viable
% of cells after 6hr (B16-F1)
Untreated
1% DMSO
5 µM adhibin
10 µM adhibin
E
Area after 2:30hr (µm²)
Control (n=71)
1% DMSO (n=72)
5 µM adhibin (n=65)
F
Circularity after 2:30hr
G
Area (µm²)
Control 0h (n=68)
Control 2:30h (n=71)
DMSO 0h (n=67)
DMSO 2:30h (n=72)
5 µM adhibin 0h (n=68)
5 µM adhibin 2:30h (n=65)
H
Area after 2:30hr (µm²)
Control (n=71)
1% DMSO (n=72)
5 µM adhibin (n=85)
I
Circularity after 2:30hr
J
BrDU
No BRDU
% B16-F1 after 24hr
Control
1% DMSO
5 µM adhibin
10 µM adhibin
K
G1 %
S %
G2 %
% B16-F1 cells
n=8264
n=7158
n=7697
n=7491
L
1% DMSO
5 µM adhibin
10 µM adhibin
25 µM adhibin
M
β-actin
1% DMSO
5 µM adhibin
-00:06 min
00:00 min
00:20 min
01:00 min
N
1% DMSO
5 µM adhibin
10 µM adhibin
Normalized event count (%)
Fluorescence intensity (A.U.)
O
F-actin
G-actin
DMSO
+adhibin
A549
MLE-12
B16-F1
P
A549
MLE-12
B16-F1
G/F actin ratio
1% DMSO
25 µM adhibin
10 µM adhibin
Q
RhoA levels (%)
total
RhoAGTP (active)
A549
B16-F1
MLE-12
R
A549
MLE-12
Active Rho-GTP, rel. (%)
Control
1% DMSO
25 µM adhibin

Supplementary Figure 4. Adhibin interferes with RhoA-mediated focal adhesion formation and maintenance. A Western blots showing Myo9a and Myo9b protein content in lysates of B16-F1 in the absence and presence of adhibin relative to GAPDH and **B** quantification of at least three independent experiments. **C** Representative bright field images of B16-F1 cells before and after adhibin treatment. Scale bars: 20 µm. **D** Viability of B16-F1 cells after 6 hours of adhibin treatment. **E** Cell area and **F** circularity of B16-F1 cells 2:30 h after seeding on laminin coated glass bottom dishes in the absence and presence of adhibin. **G** Comparison of cell area of B16-F1 cells at time point 0 h vs 2:30 h after seeding on laminin coated glass bottom dishes in the absence and presence of adhibin. **H** Cell area and **I** circularity of adherent B16-F1 cells on laminin coated glass bottom dishes 2:30 h without treatment and after treatment with 1%DMSO and 5 µM adhibin, respectively. **J** BrDU-based proliferation assay showing percentage of B16-F1 cells progressing through S-phase, 24 h after the addition of the respective treatment. **K** Cell cycle analysis of B16-F1 cells stained with DAPI and sorted via FACS. n is the number of cells analysed. **L** Representative bright field images showing primary mouse macrophages before and after 24 h treatment with adhibin. Scale bar: 20 µm. **M** FRAP of EGFP-ß-actin in lamellipodia of B16-F1 cells. White rectangle defines the photo-bleached area. Scale bar: 5 µm. Numbers in post-bleach images correspond to seconds after bleach. **N** B16-F1 cells stained for phalloidin and sorted via FACS (n=10000 events per condition). **O** Total level of F-actin and G-actin in A549, MLE-12 and B16-F1 cells in the absence and presence of adhibin. **P** G-actin/F-actin ratio in A549, MLE-12 and B16-F1 cells in the absence and presence of adhibin (18 h exposure). **Q** Pulldown assays showing changes in the level of total RhoA and RhoA•GTP after exposing cells to 25 µM adhibin overnight. **R** Relative levels of RhoA•GTP in A549 and MLE-12 cells in the absence and presence of adhibin as determined by ELISA, represented as mean ± s.e.m. n is the number of experiments. In all bar diagrams n is the number of experiments and in all box plots n is the number of cells analysed. Error bars represent mean ± S.D..

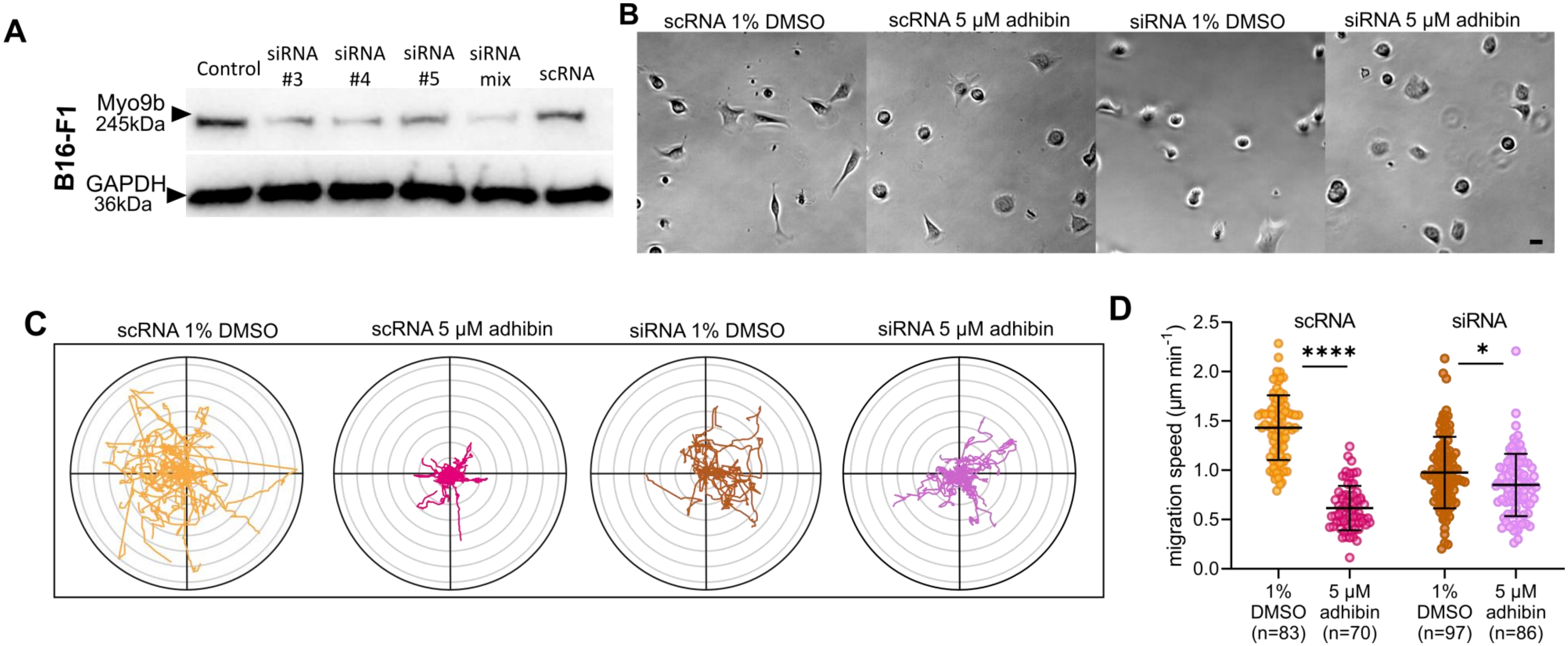

Supplementary Figure 5. B16-F1 cell phenotype after Myo9b knockdown with siRNA. A Western blot documentation of the gene silencing using different siRNAs and mixture. **B** Stills of B16-F1 cells transfected with scRNA or siRNA on laminin coated surfaces with and without adhibin treatment. Myo9b depletion causes cell roundness. Scale bar: 20 µm. **C** Radar plots from n=25 cells per condition reveal reduced migration speed of *MYO9*B silenced B16-F1 cells. **D** Migration speed distribution of B16-F1 cells transfected with scRNA or siRNA in the absence and presence of adhibin. n is the number of cells analysed. All data are represented as mean ± S.D..

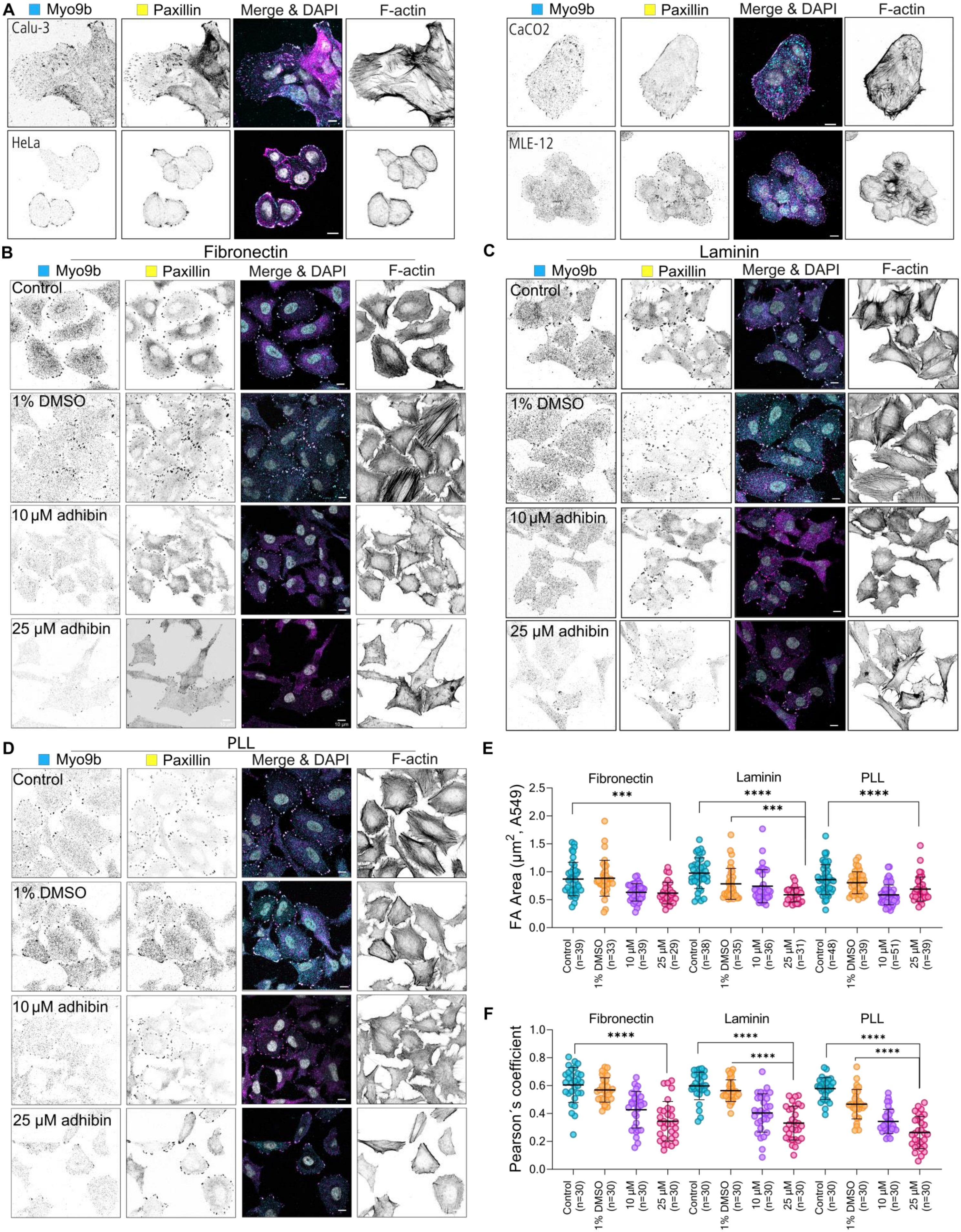
A
Myo9b
Paxillin
Merge & DAPI
F-actin
Calu-3
HeLa
CaCO2
MLE-12
B
Fibronectin
C
Laminin
Control
1% DMSO
10 μM adhibin
25 μM adhibin
D
PLL
E
FA Area (μm², A549)
***
****
F
Pearson´s coefficient
Control (n=39)
1% DMSO (n=33)
10 μM (n=39)
25 μM (n=29)
Control (n=38)
1% DMSO (n=35)
10 μM (n=36)
25 μM (n=31)
Control (n=48)
1% DMSO (n=39)
10 μM (n=51)
25 μM (n=39)
Control (n=30)
1% DMSO (n=30)
10 μM (n=30)
25 μM (n=30)

Supplementary Figure 6. Adhibin impairs cell-matrix adhesions. A Representative single Z confocal images of Calu-3, HeLa, CaCO-2 and MLE-12 cells stained for Myo9b, paxillin and phalloidin. Scale bars: 10 µm. **B-D** Representative confocal images of A549 cells seeded on laminin (**B**), fibronectin (**C**), or Poly-L-lysin (PLL) (**D**) coated surfaces stained for Myo9b, paxillin and phalloidin in the presence and absence of adhibin. Scale bars: 10 µm. **E** Quantification of focal adhesion size in A549 cells seeded on different surfaces. n is the number of cells analysed. n is the number of cells analysed. **F** Pearson's coefficient analysis of Myo9b and paxillin colocalization in A549 cells seeded on different surfaces. n is the number of focal adhesions. All data are represented as mean ± S.D..

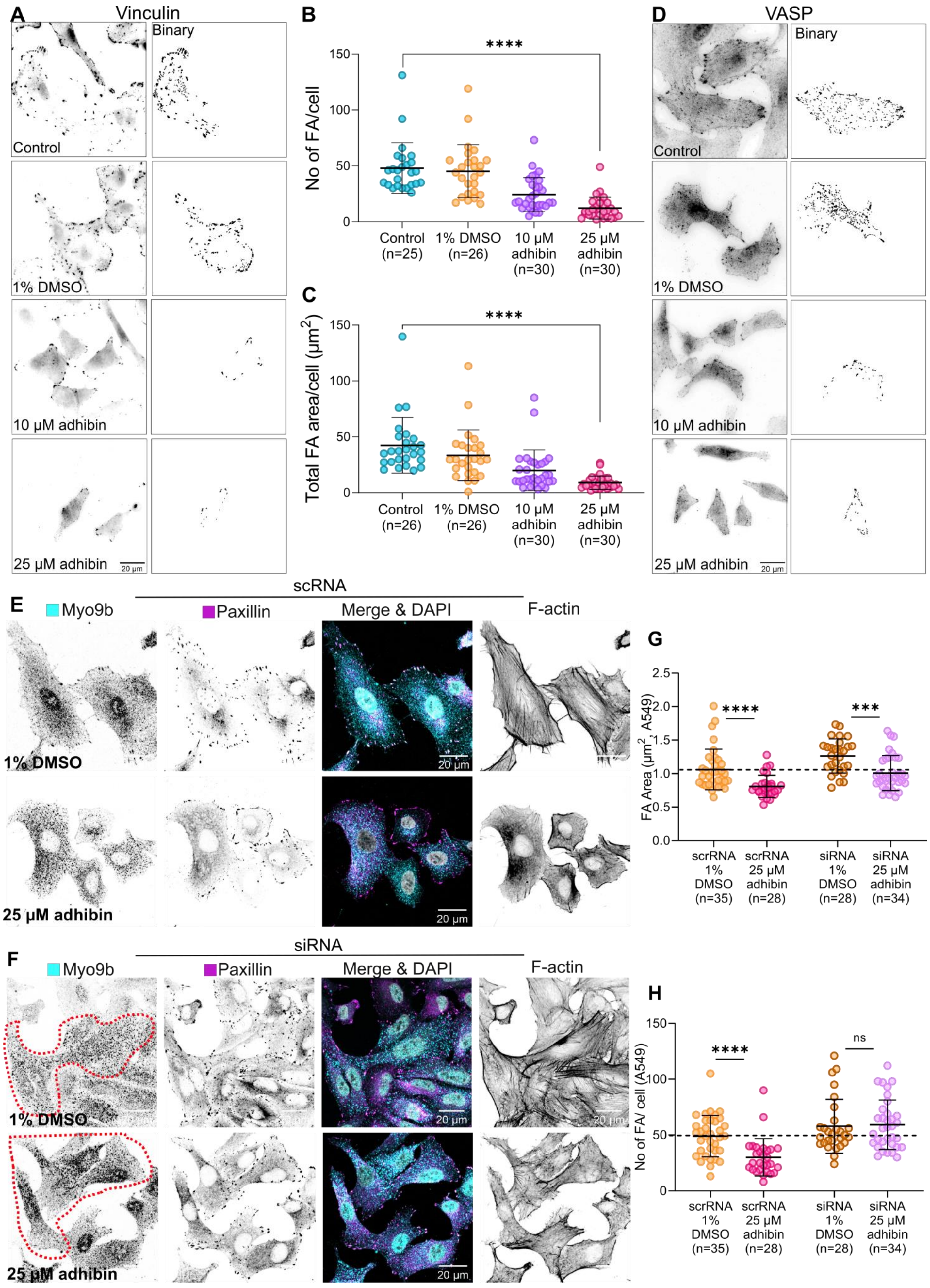

A
Vinculin
Binary
Control
1% DMSO
10 μM adhibin
25 μM adhibin
20 μm
B
No of FA/cell
****
150
100
50
0
Control (n=25)
1% DMSO (n=26)
10 μM adhibin (n=30)
25 μM adhibin (n=30)
C
Total FA area/cell (μm2)
****
150
100
50
0
Control (n=26)
1% DMSO (n=26)
10 μM adhibin (n=30)
25 μM adhibin (n=30)
D
VASP
Binary
Control
1% DMSO
10 μM adhibin
25 μM adhibin
20 μm
E
scRNA
Myo9b
Paxillin
Merge & DAPI
F-actin
1% DMSO
25 μM adhibin
20 μm
F
siRNA
Myo9b
Paxillin
Merge & DAPI
F-actin
1% DMSO
25 μM adhibin
20 μm
G
FA Area (μm2, A549)
2.5
2.0
1.5
1.0
0.5
0.0
****
***
scrRNA 1% DMSO (n=35)
scrRNA 25 μM adhibin (n=28)
siRNA 1% DMSO (n=28)
siRNA 25 μM adhibin (n=34)
H
No of FA/ cell (A549)
150
100
50
0
****
ns
scrRNA 1% DMSO (n=35)
scrRNA 25 μM adhibin (n=28)
siRNA 1% DMSO (n=28)
siRNA 25 μM adhibin (n=34)

Supplementary Figure 7. Adhibin reduces the number and area of Focal Adhesions. A Representative TIRF images and binary masks showing vinculin localization in A459 cells upon adhibin treatment. Scale bars: 20µm. **B,C** No of FA and total FA area per cell in control, 1% DMSO, 10 µM adhibin and 25 µM adhibin treated A459 cells. **D** Representative TIRF images and binary masks showing vinculin localization in A459 cells upon adhibin treatment. Scale bars: 20µm. All data are represented as mean ± S.D.. **E,F** Confocal projections showing Myo9b, paxillin and F-actin localization in A549 cells transfected with scRNA (**E**) or siRNA against Myo9b (**F**), in the presence or absence of adhibin. Scale bar: 20 µm. **G,H** Number (**G**) and area (**H**) of focal adhesions of A549 cells transfected with scRNA or siRNA against Myo9b, in the presence or absence of adhibin. n is the number of cells analysed. All data are represented as mean ± S.D..

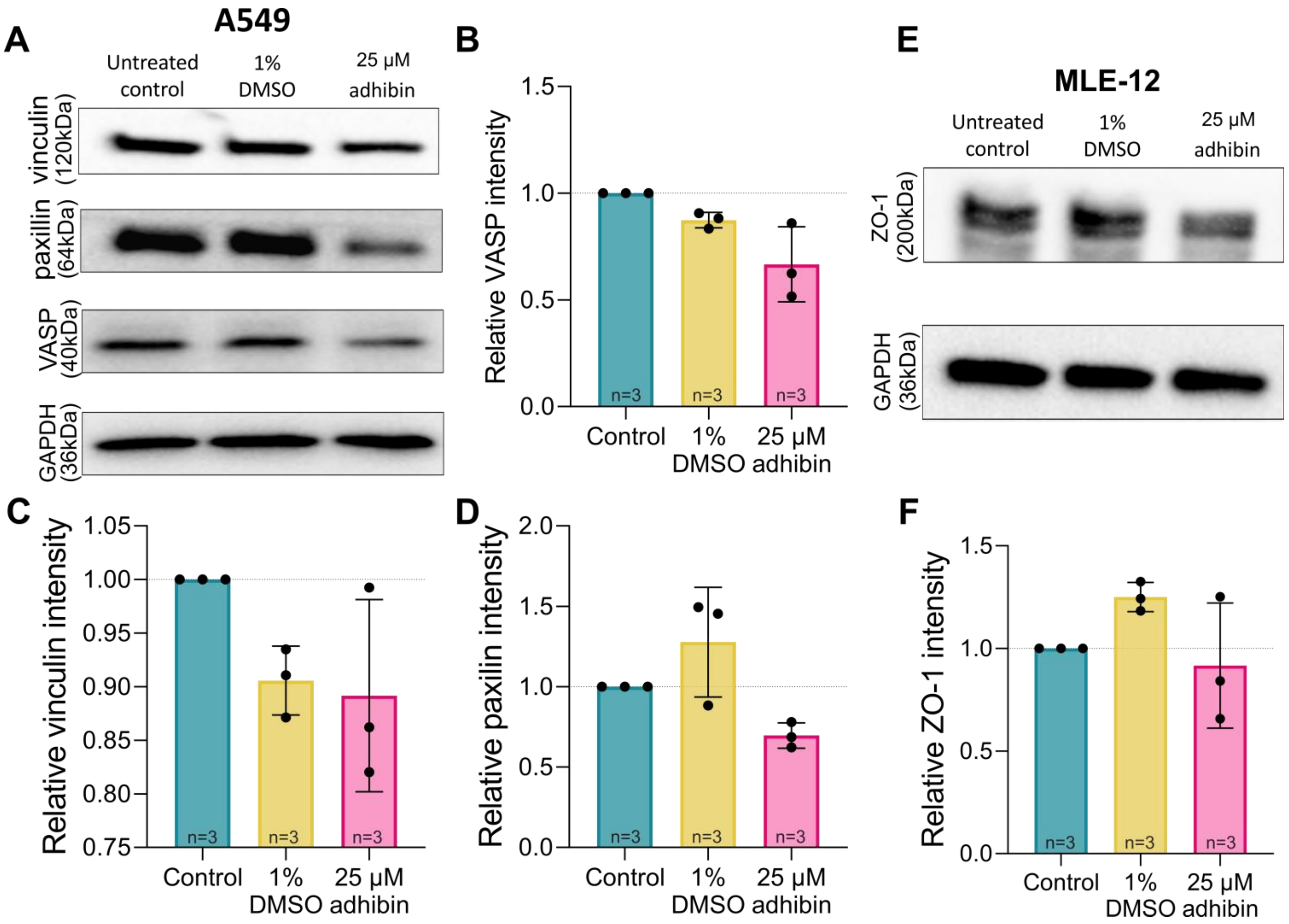

Supplementary Figure 8. Expression of marker proteins in the presence of adhibin. A Western blots comparing amount of vinculin, paxillin and VASP, between adhibin treated and A549 cells relative to GAPDH. **B,C,D** Normalized vinculin (**B**), paxillin (**C**) and VASP (**D**) intensity from 3 individual western blots. **E** Western blots comparing amount of ZO-1 between adhibin treated and MLE-12 cells relative to GAPDH. **F** Normalized ZO-1 intensity from 3 individual western blots. n is the number of experiments. All data are represented as mean ± S.D..

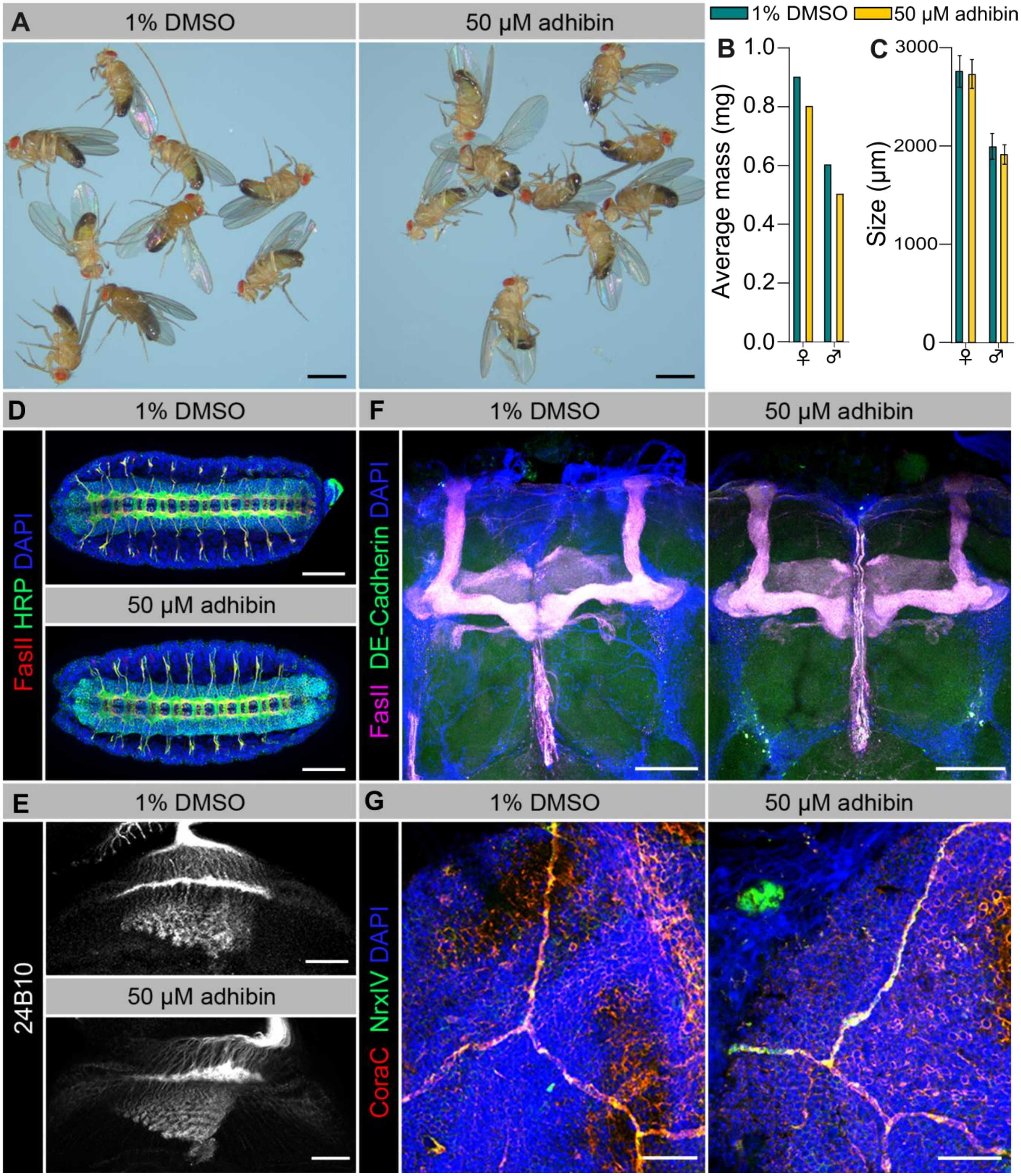

Supplementary Figure 9. Adhibin is not causing lethality in *D. melanogaster*. A Treatment with adhibin does not have any noticeable effect on the appearance of the adult flies. Scale bar: 1 mm. **B,C** Measurements of the average mass and body size. **D** Confocal images of the nervous system of *Drosophila* embryo stained with HRP (green, ubiquitous CNS marker), N-CAM homolog Fasciclin II (FasII, red, longitudinal axons), and DAPI (blue, nuclei). Scale bar: 50 µm. **E** Confocal images of the eye-brain complexes from third-instar larvae

stained with 24B10 (grey) to visualize photoreceptor cell axonal projections reveal that both control and adhibin-treated animals display typical axonal projection patterns. Scale bar: 20 µm. **F** Administering Adhibin throughout the entire developmental and lifespan stages does not affect brain compartmentalization. Adult brains stained with FasII (magenta, labels α/β and γ lobes in the learning and memory center), DE-Cadherin (green, cell adhesion marker) and DAPI (blue, nuclei). Scale bar: 50 µm. **G** Based on the staining of adult brains with tight junction markers Coracle C (CoraC, red) and Neurexin IV (NrxIV, green), adhibin administration does not influence tight junction formation in the blood-brain barrier of Drosophila. Tight junctions are represented as condensed and distinct strands. DAPI (blue, nuclei). Scale bar: 20 µm. All data are represented as mean ± S.D..

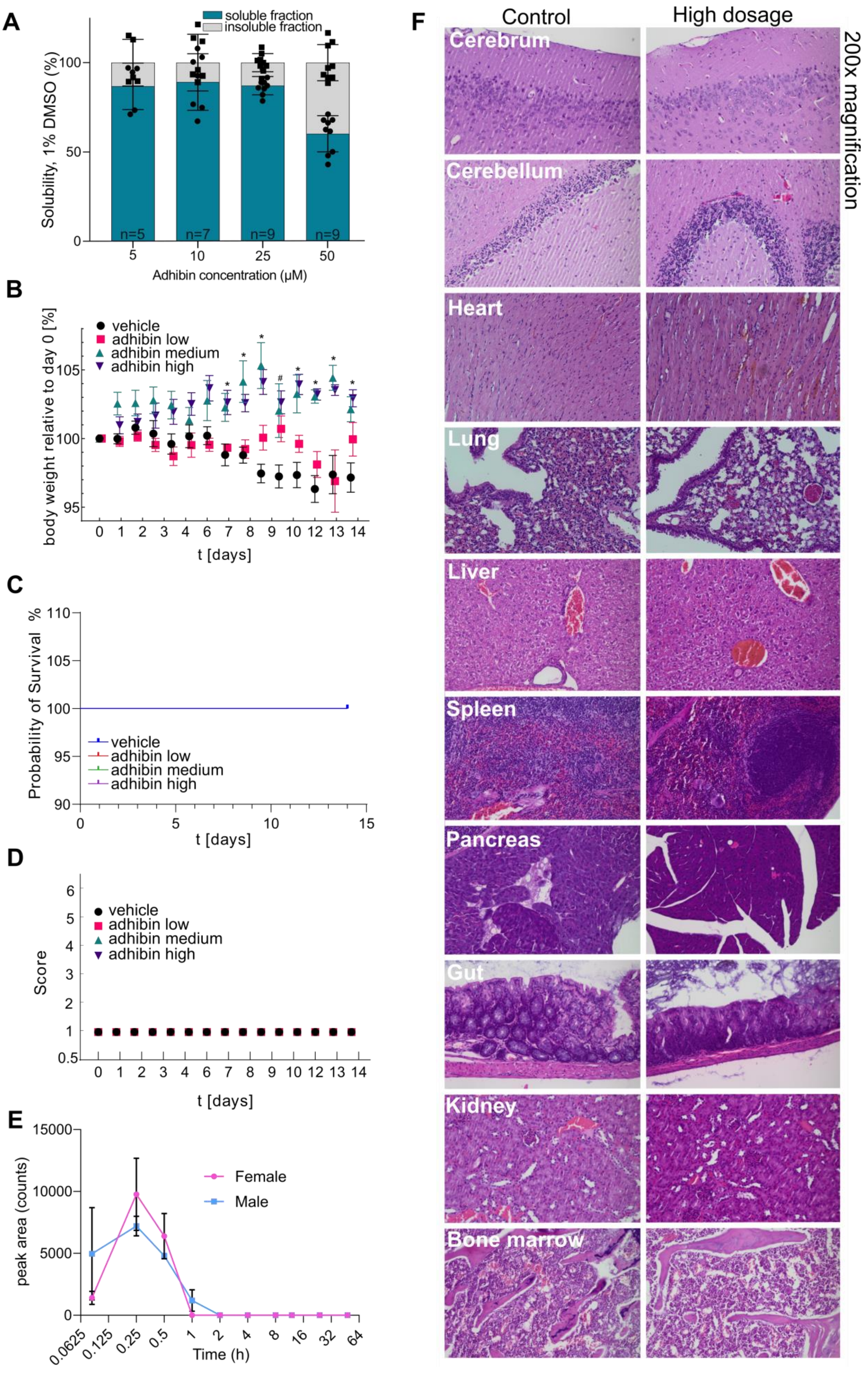

A
soluble fraction
insoluble fraction
Solubility, 1% DMSO (%)
n=5
n=7
n=9
n=9
Adhibin concentration (μM)
B
vehicle
adhibin low
adhibin medium
adhibin high
body weight relative to day 0 [%]
t [days]
C
Probability of Survival %
vehicle
adhibin low
adhibin medium
adhibin high
t [days]
D
vehicle
adhibin low
adhibin medium
adhibin high
Score
t [days]
E
peak area (counts)
Female
Male
Time (h)
F
Control
High dosage
200x magnification
Cerebrum
Cerebellum
Heart
Lung
Liver
Spleen
Pancreas
Gut
Kidney
Bone marrow

Supplementary Figure 10. Adhibin is not causing lethality in *M. musculus*. A Solubility of adhibin in DMEM containing 1% FBS and 1% DMSO. **B** Relative body weight of mice after adhibin administration (0.142, 0.047, and 0.016 mg/kg body weight) at day 0. n is the number of experiments. **C** Kaplan-Meyer data showing probability of survival over a 14 days' period after adhibin administration. **D** Clinical scoring of mice after adhibin administration. n is the number of experiments. All data are represented as mean ± S.D.. **E** Normalized peak area of adhibin in the serum at defined time points after intraperitoneal injection. **F** HE stainings of cerebrum, cerebellum, heart, lung, liver, spleen, pancreas, gut, kidney and bone marrow in control and drug treated mice at highest tolerable doses as imposed by the Specialised Department of Animal Welfare Service of Lower Saxony.

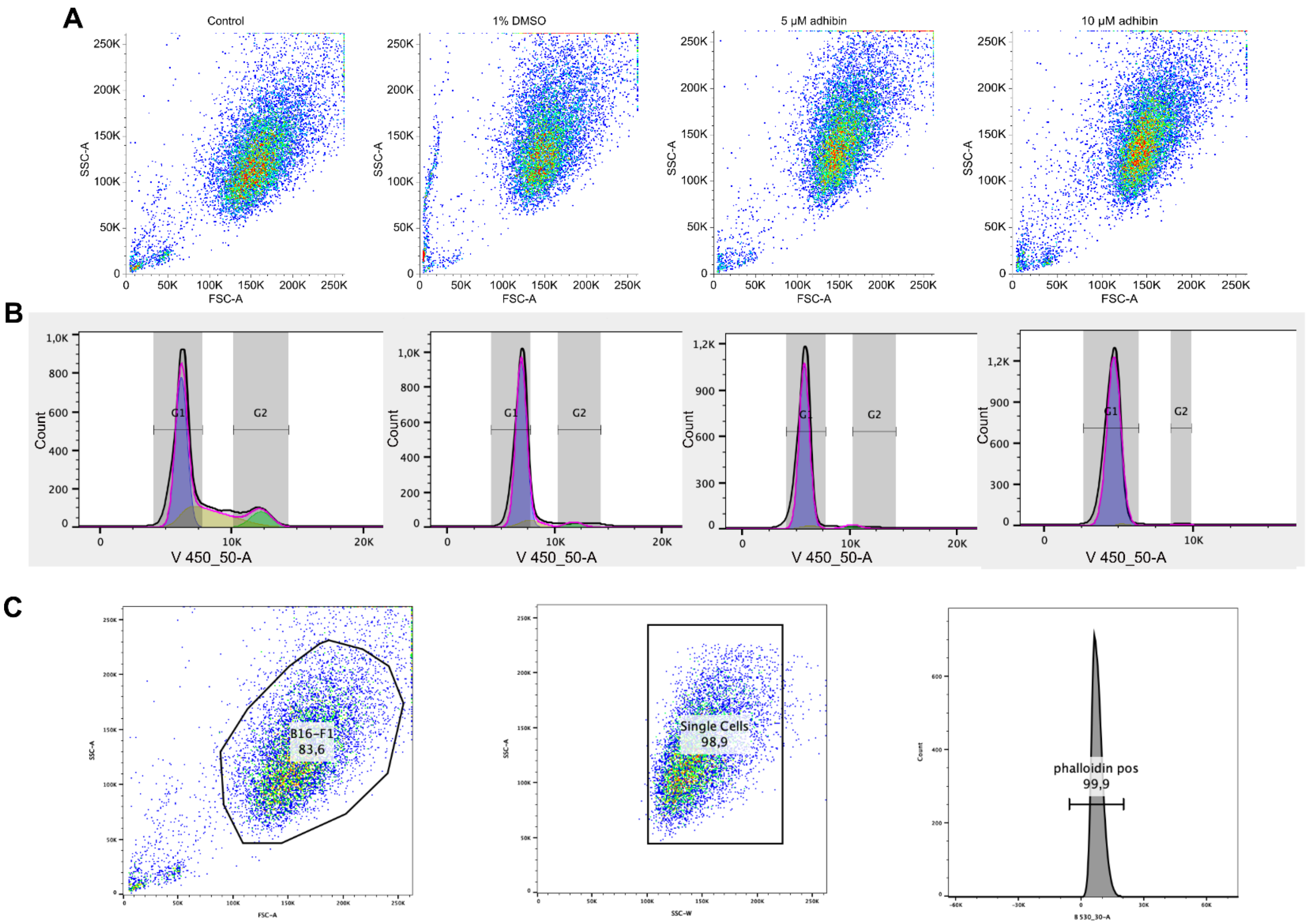

Supplementary Figure 11. FACS Data and gating explanation. A Histograms showing the populations of control, 1% DMSO, 5 µM and 10 µM adhibin treated B16-F1 cells. **B** Cell cycle analysis of A. V 450_50-A: cells stained with DAPI (refers to Supplementary Figure 4K) . **C** Gating explanation: Gating of cells based on scatter properties (FSC/SSC), exclusion of doublets (SSC-A/SSC-W), gating on phalloidin positive cells (phalloidin atto488). B 530_30-A: cells stained with phalloidin. 10.000 total events were analyzed per sample. (refers to Supplementary Figure 4N).

SUPPLEMENTARY TABLES AND LEGENDS

Supplementary Table 1. Selectivity of compounds.

Inhibitor	Parameter	*Dd*Myo2	*Hs*NM2A	Sk muscle Myo2	βcard. muscle Myo2	*Dd*Myo5b	*Ce*Myo9	*Rn*Myo9b
Adhibin (**12**)	K_D (µM)	14.9 ± 3.2	*No effect	*No effect	*No effect	1.5 ± 0.5	n.d.	n.d.
	IC_{50} (µM)	37.7 ± 9.4				144.6 ± 26.3	2.5 ± 0.2	2.6 ± 0.2
Analog (**5**)	K_D (µM)	7.3 ± 4.1				33.2 ± 5.2	n.d.	n.d.
	IC_{50} (µM)	22.6 ± 7.5				91.2 ± 18.4	n.d.	2.0 ± 0.3

* up to 50 µM concentrations

Supplementary Table 2. Data collection and refinement statistics (molecular replacement).

	*Dd*Myo–2 + adhibin analogue 5
**Data collection**	
Space group	$C222_1$
Cell dimensions	
a, *b*, *c* (Å)	90.93, 144.22, 156.94
α,β,γ (°)	90, 90, 90
Resolution (Å)	45.47 – 3.2 (3.31 – 3.2) *
R_{sym} or R_{merge}	0.25 (1.58)
$I / \sigma I$	9.51 (1.75)
Completeness (%)	99.87 (99.94)
Redundancy	12.1
Refinement	
Resolution (Å)	45.47 – 3.2
No. reflections	17,390
R_{work} / R_{free}	21.8 / 26.3
No. atoms	5885
Protein	5769
Ligand/ion	91
Water	25
B–factors	
Protein	83.93
Ligand/ion	77.28
Water	57.47
R.m.s. deviations	
Bond lengths (Å)	0.003
Bond angles (°)	0.49

*Number of xtals for each structure should be noted in footnote. *Values in parentheses are for highest–resolution shell.

[AU: Equations defining various *R*–values are standard and hence are no longer defined in the footnotes.]
[AU: Ramachandran statistics should be in Methods section at the end of Refinement subsection.]

Supplementary Table 3. Effect of adhibin on viability.

		Cell line					Organism			
**Inhibitor**	**Parameter**	**A549**	**MLE-12**	**Calu-3**	**HepG2**	**B16-F1**	***E.coli***	***D. discoideum***	***D. melanogaster***	***M. musculus***
Adhibin	EC50 (µM)	36.7 ± 2.5	27.9 ± 2.9	> 70.0	> 40.0	7.94 ± 3.34	No effect	#No effect	*No effect	No effect
Analog (**5**)	EC_{50} (µM)	47.7 ± 12.6	31.6 ± 5.0	25.3 ± 9.1	> 50.0	n.d.	n.d.	n.d.	n.d.	n.d.
PBP	EC_{50} (µM)	1.6 ± 1.3	3.6 ± 1.9	n.d.	0.03 ± 0.01	n.d.	n.d.	n.d.	n.d.	n.d.
PClP	EC_{50} (µM)	2.7 ± 0.9	1.9 ± 1.3	n.d.	0.8 ± 0.2	n.d.	n.d.	n.d.	n.d.	n.d.

# up to 50 µM concentrations
* up to 50 µM concentrations no effect on mass and size
^{X} up to 0.5 µM concentration in blood no lethality

Supplementary Table 4. Drug uptake in *D. melanogaster*.

Sample-No.	Concentration adhibin (µM)	Number of animals	Status of animals	Adhibin [pmol/sample]
1 A	Control, 0 µM	25	adult	No Peak
2 A	1% DMSO, 0 µM	25	adult	-
3 A	25 µM	25	adult	0.532
4 A	50 µM	25	adult	4.06
5 A	100 µM	25	adult	17.5
6 T	Control, 0 µM	25	adult	0.00243
7 T	1% DMSO, 0 µM	25	adult	-
8 T	25 µM	25	adult	0.149
9 T	50 µM	25	adult	2.94
10 T	100 µM	25	adult	(0.0357)
11 L3	Control, 0 µM	10	larva	0.000424
12 L3	1% DMSO, 0 µM	10	larva	-
13 L3	25 µM	10	larva	1.28
14 L3	50 µM	10	larva	2.45
15 L3	100 µM	10	larva	8.51

A: adult flies licking the surface of the food; L3: larvae eating food supplemented with the drug in micromolar amounts as specified; T: adult flies that arose from L3. Bold numbers show the increase in drug uptake with increasing amounts of administered drug.

Supplementary Table 5. Detailed information about Myosin purification.

Name	Figure	Organism	Expression system	Purification tag	Reference
**Skeletal heavy meromyosin, *(skHMM)***	1D, 1E	*O. cuniculus*	Skeletal muscle	None, native	[1]
***Ss*β-cardMyo2**	1E	*S. scrofa*	heart	None, native	[2]
***Dd*Myo2**	1E, 1G, 1I	*D. discoideum*	*D. discoideum*	N-term. 8-His	[3]
***Dd*Myo5a**	1E, 1G, 1I	*D. discoideum*	*D. discoideum*	N-term. 8-His	Same protocol as for *Dd*Myo5b in reference [4]
***Dd*Myo5b**	1E, 1G, 1I	*D. discoideum*	*D. discoideum*	N-term. 8-His	[4]
***Rn*Myo9b**	1B, 1C, 1E,1I	*R. norvegicus*	*Sf9/baculovirus*	N-term. 6-His	[5]
***Ce*Myo9**	1C, 1G	*C. elegans*	*Sf9/baculovirus*	C-term. Avi/FLAG	[6]
***Hs*NM2a**	1F, 1I	*H. sapiens*	*Sf9/baculovirus*	N-term. 8-His	[7]
***Hs*NM2b**	1F	*H. sapiens*	*Sf9/baculovirus*	N-term. 8-His	[8]
***Hs*NM2c**	1F	*H. sapiens*	*Sf9/baculovirus*	N-term. 8-His	[7]

SUPPLEMENTARY METHODS

Reagent and resource information

	SOURCE	IDENTIFIER
**Antibodies**		
anti-Myo9a	Thermo Fisher Scientific	Cat# PA5-59055; RRID:AB_2644379
anti-Myo9b	Proteintech	Cat# 12432-1-AP; RRID:AB_2148635
anti-paxillin	BD Biosciences	Cat# 610051; RRID:AB_397463
anti-ZO1	Thermo Fisher Scientific	Cat# 40-2200; RRID:AB_2533456
anti-connexin43	BD Biosciences	Cat# 610062; RRID:AB_397474
anti-pMLC2	Cell signalling	Cat# 3671; RRID:AB_330248
anti-NMIIA	Biolegend	Cat# 909801; RRID:AB_2565100
anti-NMIIB	Biolegend	Cat# 909901; RRID:AB_2749903
anti- phospho-MYPT1 (Thr850)	Sigma-Aldrich	Cat# 36-003; RRID:AB_310812
anti-pan Actin	Abcam	Cat# ab119952
anti-vinculin	Sigma-Aldrich	Cat# V9131; RRID:AB_477629
anti-VASP	from Jan Faix, Damiano-Guercio et al., 2020	N/A
anti-GAPDH	Sigma-Aldrich	Cat# CB1001-500UG
anti-BrDU	Invitrogen	Cat# MA3-071; RRID:AB_10986341
anti-RhoA	Santa Cruz Biotechnology	Cat# 26C4

anti-24B10	Developmental Studies Hybridoma Bank	24B10; RRID: AB_528161
anti-CoraC	Developmental Studies Hybridoma Bank	C615.16; RRID: AB_1161644
anti-FasII	Developmental Studies Hybridoma Bank	1D4 anti-Fasciclin II, RRID: AB_528235
anti-DE-Cadherin	Developmental Studies Hybridoma Bank	DCAD2, RRID: AB_528120
anti-NrxIV	from Christian Klämbt	N/A
Rabbit IgG (H&L) Secondary Antibody Peroxidase Conjugated Pre-adsorbed	Rockland	Cat# 611-103-122
AlexaFluor555-conjugated goat anti-rabbit	Thermo Fisher Scientific	Cat# A-21428; RRID:AB_2535849
AlexaFluor488-conjugated goat anti-mouse	Thermo Fisher Scientific	Cat# A-11029; RRID:AB_2534088
AlexaFluor488-conjugated goat anti-rat	Thermo Fisher Scientific	Cat# A-11006; RRID:AB_2534074
AlexaFluor488-conjugated goat anti-rabbit	Thermo Fisher Scientific	Cat# A-11008; RRID:AB_143165
IgG1 Cy3 goat anti-mouse	Jackson ImmunoResearch Laboratory	Cat# 115-165-205
AlexaFluor633-conjugated phalloidin	Thermo Fisher Scientific	Cat# A12379; RRID:AB_2534069
Atto633- conjugated phalloidin	ATTO-TECH	Cat# AD 633-8
Atto488- conjugated phalloidin	ATTO-TECH	Cat# AD 488-8
iFlour™488 anti-rabbit	ATT Bioquest	Cat# 16608
Stabilized peroxidase conjugated goat anti-rabbit, HRP conjugated	Invitrogen	Cat# 32460; RRID: AB_1185567
Stabilized peroxidase conjugated goat anti-mouse, HRP conjugated	Invitrogen	Cat# 32430; RRID: AB_1185566

**Chemicals**		
3,6-Dibromo-1-hydroxymethyl-9H-carbazole **Adhibin** (12)	This paper	N/A
3,4-Dibromo-1-methoxy-6-methyl-9H-carbazole (4)	This paper	N/A
3,4-Dibromo-6-methyl-9H-carbazol-1-ol (5)	This paper	N/A
Methyl 3,6-dibromo-9H-carbazol-1-carboxylate (9)	This paper	N/A
3,6-Dibromo-9H-carbazol-1-carboxylic acid (10)	This paper	N/A
Dulbecco´s Modified Eagle Medium **(DMEM)**	Sigma-Aldrich	Cat# D5796
Fetal Bovine Serum	Biowest	Cat# S1400-500
Penicillin streptomycin	Gibco	Cat# 15140122
Laminin	Sigma-Aldrich	Cat# 11243217001
Fibronectin	Sigma-Aldrich	Cat# 11051407001
Poly-L-lysine	Sigma-Aldrich	Cat# P1524
4–20% Mini-PROTEAN TGX Precast Protein Gels	BioRad	Cat# 4561096
Bovine Serum Albumin	Sigma-Aldrich	Cat# A2153
BrDU (5-Bromo-2´-Deoxyuridine)	Thermo Fisher Scientific	Cat# B23151
Prolong Gold antifade mountant with DAPI	Invitrogen	Cat# P36935
Formaldehyde, 16%	Polysciences Inc	Cat# 18814-20
Normal Goat Serum	Abcam	Cat# ab7481
DAPI	Sigma Aldrich	Cat# D9542-10MG
L-WRN conditioned medium	Miyoshi and Stappenbeck, 2013	N/A
Advanced DMEM/F12	Invitrogen	Cat# 12634-028
HEPES 1M	Invitrogen	Cat# 15630-056
GlutaMAX-I	Invitrogen	Cat# 35050-079
Primocin	Invitrogen	Cat# ant-pm-1
N2 supplement	Invitrogen	Cat# 17502-048
B27 supplement	Invitrogen	Cat# 17504-044
N-Acetylcysteine	Sigma-Aldrich	Cat# A9165-5G
human recombinant EGF	Invitrogen	Cat# PMG8043
A-83-01	Tocris	Cat# 2939
SB202190	Tocris	Cat# 1264

Nicotinamide	Sigma-Aldrich	Cat# N0636
[Leu15]-Gastrin I	Sigma-Aldrich	Cat# G9145
CHIR99021	Sigma-Aldrich	Cat# SML1046-5MG
Y-27632 dihydrochloride	Tocris	Cat# 1254
**Commercial assays**		
RNeasy Mini Kit	Qiagen	Cat# 74104
QuantiNova Reverse Transcription Kit	Qiagen	Cat# QT02589307
G-LISA RhoA Activation Assay Biochem Kit	Cytoskeleton	Cat# BK124
RhoA Pull-Down Activation Assay Biochem Kit (Bead Pull-Down Format)	Cytoskeleton	Cat# BK036
**Deposited Data**		
Crystal structure of adhibin analogue-bound myosin-2	Protein Data Bank (PDB)	6Z2S
**Biological samples**		
Primary macrophages	BALB/cJRj	N/A
Human colonoids	Intestinal biopsies, transverse colon, healthy volunteered donor	N/A
**Cell lines**		
A549	ATCC	CCL-185; RRID: CVCL_0023
B16-F1	ATCC	CRL-6323; RRID:CVCL_0158
MLE-12	ATCC	CRL-2110; RRID:CVCL_3751
CaCo-2	ATCC	HTB-37; RRID:CVCL_0025
Calu-3	ATCC	HTB-55; RRID:CVCL_0609

NIH/3T3	ATCC	CRL-1658; RRID:CVCL_0594
HeLa	ATCC	CRM-CCL-2 RRID:CVCL_0030
**Organisms/strains**		
Drosophila Melanogaster: Oregon R, wild-type strain	Bloomington *Drosophila* Stock Center	BDSC: 5 FBgn0003996
Mus musculus: BALB/cJRj	The Jackson Laboratory	JAX:000651; RRID:IMSR
**Oligonucleotides**		
TCGTCTCAAAGATATCAGTAGC	*Hs myo9a* forward primer	N/A
CTTTCCCATTGATCTACGA	*Hs myo9a* reverse primer	N/A
TTGTTTATCAAGAGCATAATGA	*Mm myo9a* forward primer	N/A
AGAGGTTTCTTTGTTATCTGG	*Mm myo9a* reverse primer	N/A
TATTATTTGTTACTTGGGGTCA	*Hs myo9b* forward primer	N/A
ATCTTCAATCTTCAAGTTATGC	*Hs myo9b* reverse primer	N/A
TCCAAGTCAACTACCTAGAAAA	*Mm Myo9b* forward primer	N/A
AGCAGCAGATAATAAAACACAT	*Mm Myo9b* reverse primer	N/A
GTCTCCTTTGAGCTGTTTG	*Hs PPIA* forward primer	N/A
ATAAACCCTGGAATAATTCTGT	*Hs PPIA* reverse primer	N/A
TGCACTGCCAAGACTGAATG	*Mm Ppia* forward primer	N/A

CCATGGCTTCCACAATGTTC	*Mm Ppia* reverse primer	N/A
GACCTCAAACTGGCTCCTAATC	Ki-67 forward primer	N/A
GCTGCCAGATAGAGTCAGAAAG	Ki-67 reverse primer	N/A
CTACCACAACCTCTTCTCCC	Paxillin forward primer	N/A
AAAGCACTCAGGATGCCA	Paxillin reverse primer	N/A
AAACCACCTCTGCCTGAAG	Vinculin forward primer	N/A
ACAACACCTATACCCACCTC	Vinculin reverse primer	N/A
AGAACAGCACAACCTTGCC	VASP forward primer	N/A
CAATTCCTTCTTCACCTCTTCC	VASP reverse primer	N/A
AGAAAAGTGGACCCCAGAAG	RhoA forward primer	N/A
CAAAAACCTCTCTCACTCCATC	RhoA reverse primer	N/A
ACTCTTCCAGCCTTCCTTC	β-actin forward primer	N/A
TCTCCTTCTGCATCCTGTC	β-actin reverse primer	N/A
CGGAACAGTTCAAGGACCAG	NMIIA (MHY9) forward primer	N/A
GAGTCACGACAAATGGCAG	NMIIA (MHY9) reverse primer	N/A
CAGCACATGACGGAGGTTGT	TP53 forward primer	N/A
TCATCCAAATACTCCACACGC	TP53 reverse primer	N/A
TGCTGAAAGACATTATGACACC	pTEN forward primer	N/A

TCATTACACCAGTTCGTCCC	pTEN reverse primer	N/A
TTGAGCAAATGAGGCGAC	PIK3CA forward primer	N/A
GCAGAGGACATAATTCGACAC	PIK3CA reverse primer	N/A
ACAGAGAGTGGAGGATGCTTT	KRAS forward primer	N/A
TTTCACACAGCCAGGAGTCTT	KRAS reverse primer	N/A
AGGCACGAGTAACAAGCTCAC	EGFR forward primer	N/A
ATGAGGACATAACCAGCCACC	EGFR reverse primer	N/A
GTGTCCATTGAGGGTATCCACC	KEAP1 forward primer	N/A
GCTCAGCGAAGTTGGCGAT	KEAP1 reverse primer	N/A
AAAGCCTACATCGCTACCC	PTPRD forward primer	N/A
ACATTTCTCTCTGCCCATTTC	PTPRD reverse primer	N/A
TCAAACATCAACAACAGGGAC	BRAF forward primer	N/A
ATCTCTTCATGGCTTTTGGAC	BRAF reverse primer	N/A
TCAGCACAGTTCGTGAGGTG	CDK4 forward primer	N/A
GTCCATCAGCCGGACAACAT	CDK4 reverse primer	N/A
**Recombinant DNA**		
pEGFC1-dtomato-2xrRBD.	This paper	N/A
pEGFC2-β-actin	This paper	N/A

pEGFPN1-Paxillin	Jan Faix (Hannover Medical School)	N/A
mScarletN1-LifeAct	Jan Faix (Hannover Medical School)	N/A
pEGFPC1-Myr5	van den Boom et al.	N/A
**Software and algorithms**		
Fiji	Schindelin et al.	https://hpc.nih.gov/apps/Fiji
Excel	Microsoft	https://www.microsoft.com/en-us/microsoft-365/excel
PowerPoint	Microsoft	https://www.microsoft.com/en/microsoft-365/powerpoint
XDS	XDS Program Package	https://xds.mr.mpg.de/
AIMLESS	Ccp4 software suite	https://www.mrc-lmb.cam.ac.uk/harry/pre/aimless.html
Phaser	McCoy et al.	N/A
Coot	Emsley et al.	https://www2.mrc-lmb.cam.ac.uk/personal/pemsley/coot/
Phenix.refine	Afonine et al.	https://phenix-online.org/download/
Modeller 9.16	Fiser et al.	https://salilab.org/modeller/
Autodock4	Morris et al.	https://autodock.scripps.edu/download-autodock4/
MacroModel Release 2019-3	Schrödinger Maestro Harder et al.	https://newsite.schrodinger.com/platform/products/macromodel/
Zen	ZEISS	https://www.zeiss.com/microscopy/de/produkte/software/zeiss-zen.html

Sigma Plot	Systat Software GmbH	https://systatsoftware.com/sigmaplot
FlowJo™ Software	BD Life Sciences	https://www.bdbiosciences.com/en-de/products/software/flowjo-v10-software
Prism 8	GraphPad Software	https://www.graphpad.com
Origin 7/ Origin 8	Origin Lab	https://www.originlab.com
DiaTrack 3.0 software	Vallotton et al.	N/A
Inkscape Project	Inkscape	https://inkscape.org

Compound syntheses

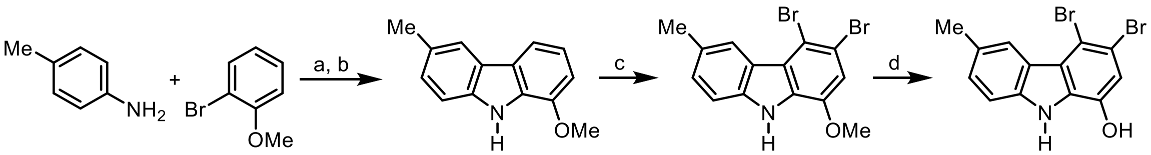

Synthesis of **4** and **5**. a: 1.2 equiv. **1**, 6 mol% $Pd(OAc)_2$, 6 mol% BINAP, 1.2 equiv. Cs_2CO_3, toluene, reflux, 48 h, 98%; b: 10 mol% $Pd(OAc)_2$, air, HOAc, 80 °C, 24 h, 63%; c: 2.0 equiv. NBS, cat. HBr, MeCN, 0 °C to 23 °C, 40 min, 86%; d: 20 equiv. BBr_3, $(CH_2Cl)_2$, 84 °C, 30 min, 99%.

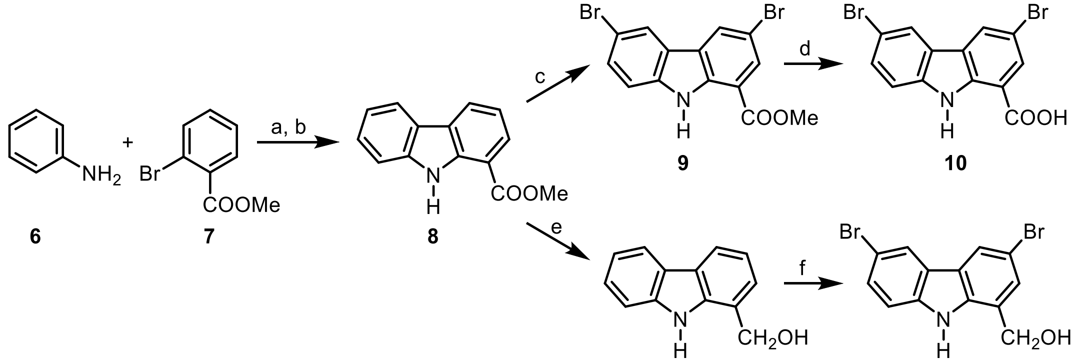

Synthesis of **9**, **10**, and adhibin (**12**). a: 1.2 equiv. **6**, 6 mol% $Pd(OAc)_2$, 6 mol% BINAP, 1.2 equiv. Cs_2CO_3, toluene, reflux, 45 h, 82%; b: 1.5 equiv. $Pd(OAc)_2$, HOAc, reflux, 2 h, 64%; c: 5.0 equiv. pyridinium tribromide, EtOH, 23 °C, 6 h, 98%; d: 1. 17 equiv. KOH, THF–H_2O (2:1), 23 °C, 24 h, 2. 2 N HCl, 100%; e: 2.5 equiv. DIBAL-H, CH_2Cl_2, –78 °C to 23 °C, 1 h, 97%; f: 3.0 equiv. pyridinium tribromide, EtOH, 23 °C, 5 h, 92%.

General: All reactions were carried out using dry solvents in oven-dried glassware under an argon atmosphere, unless stated otherwise. Silica gel 60 M, 0.04–0.063 mm, was purchased from Macherey-Nagel. Melting points were measured on an Electrothermal IA9100 melting point apparatus. UV spectra were recorded on a Varian Cary 5e spectrometer. Fluorescence spectra were measured on a Varian Cary Eclipse spectrometer. IR spectra were recorded on a Thermo Nicolet Avatar 360 FT-IR spectrometer using the ATR method (Attenuated Total Reflection). NMR spectra were recorded on Bruker AC 300-P and Bruker DRX 500 spectrometers, chemical shifts δ are reported in ppm with the solvent signal as internal standard. Coupling constants were determined

assuming first-order spin-spin coupling. The multiplicity of the carbon signals was determined with the help of DEPT 135 spectra. Assignment of the ^{1}H NMR and ^{13}C NMR signals was achieved using the 2D NMR methods COSY, HSQC, HMBC, and NOESY. Mass spectra were recorded on a Finnigan MAT-95 spectrometer (electron impact, 70 eV) or by GC-MS coupling using an Agilent Technologies 6890N GC System equipped with a 5973 Mass Selective Detector (electron impact, 70 eV). HRMS were recorded on a Finnigan MAT-95 spectrometer (electron impact, 70 eV). Elemental analyses have been carried out on a Euro Vector EuroEA 3000 CHNS elemental analyzer.

2-Methoxy-*N*-(*p*-tolyl)aniline

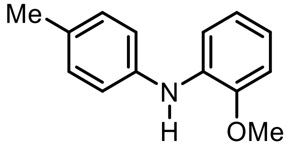

p-Toluidine (**1**) (206 mg, 1.92 mmol) and then *o*-bromoanisole (**2**) (300 mg, 1.60 mmol) were added to a suspension of palladium(II) acetate (21.6 mg, 0.096 mmol), *rac*-BINAP (59.9 mg, 0.096 mmol), and cesium carbonate (627 mg, 1.92 mmol) in toluene (15 mL). The mixture was heated under reflux for 48 h and then filtered with ethyl acetate (250 mL) through a short path of Celite and silica gel. Removal of the solvent in vacuum and purification of the crude product by column chromatography on silica gel (petroleum ether/ethyl acetate, 9:1) afforded 2-methoxy-*N*-(*p*-tolyl)aniline as a brownish oil; yield: 336 mg (1.58 mmol, 98%).

IR (ATR): ν = 3410, 3018, 2936, 2830, 1597, 1513, 1457, 1296, 1232, 1113, 1026, 807, 736 cm^{-1}.

^{1}H NMR (500 MHz, $CDCl_3$): δ = 2.31 (s, 3 H), 3.89 (s, 3 H), 6.40 br s, 1 H), 6.82–6.88 (m, 3 H), 7.06–7.11 (m, 4 H), 7.22 (dd, *J* = 7.6, 1.8 Hz, 1 H).

^{13}C NMR and DEPT (125 MHz, $CDCl_3$): δ = 20.72 (CH_3), 55.58 (CH_3), 110.39 (CH), 113.93 (CH), 119.43 (CH), 119.69 (2 CH), 120.84 (CH), 129.79 (2 CH), 131.20 (C), 133.60 (C), 139.74 (C), 147.93 (C).

EI-MS: *m*/*z* (%) = 213 (94) [M^+], 198 (38), 183 (100), 154 (15).

Elemental analysis (%) calcd. for $C_{14}H_{15}NO$: C 78.84, H 7.09, N 6.57; found: C 78.94, H 7.07, N 6.74.

1-Methoxy-6-methyl-9*H*-carbazole (3)

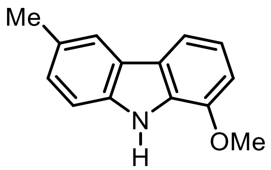

Palladium(II) acetate (21.4 mg, 0.095 mmol) was added to a solution of 2-methoxy-*N*-(*p*-tolyl)aniline (204 mg, 0.957 mmol) in acetic acid (10 mL). The mixture was heated at 80 °C under air for 24 h. After cooling to room temperature, the mixture was filtered with dichloromethane (150 mL) through a short path of Celite and silica gel. Removal of the solvent in vacuum and purification of the crude product by column chromatography on silica gel (petroleum ether/ethyl acetate, 9:1) provided 1-methoxy-6-methyl-9*H*-carbazole (**3**) as a light yellow solid; yield: 127 mg (0.601 mmol, 63%); mp: 195–196 °C.

UV (MeOH): λ = 224, 243, 252, 260 (sh), 281, 291, 327, 341 nm.

IR (ATR): ν = 3411, 3016, 2929, 2841, 1576, 1449, 1392, 1317, 1296, 1254, 1234, 1094, 1027, 799, 782, 746, 729, 696, 602 cm^{-1}.

^{1}H NMR (500 MHz, $CDCl_3$): δ = 2.52 (s, 3 H), 4.00 (s, 3 H), 6.88 (d, *J* = 7.8 Hz, 1 H), 7.13 (t, *J* = 7.8 Hz, 1 H), 7.22 (dd, *J* = 8.2, 1.3 Hz, 1 H), 7.34 (d, *J* = 8.2 Hz, 1 H), 7.64 (d, *J* = 7.8 Hz, 1 H), 7.84 (d, *J* = 0.5 Hz, 1 H), 8.14 (br s, 1 H).

^{13}C NMR and DEPT (125 MHz, $CDCl_3$): δ = 21.43 (CH_3), 55.48 (CH_3), 105.69 (CH), 110.55 (CH), 112.78 (CH), 119.46 (CH), 120.41 (CH), 123.81 (C), 124.15 (C), 127.05 (CH), 128.64 (C), 130.07 (C), 137.40 (C), 145.64 (C).

EI-MS: *m/z* (%) = 211 (100) [M^+], 196 (88), 168 (71), 153 (11).

Elemental analysis (%) calcd. for $C_{14}H_{13}NO$: C 79.59, H 6.20, N 6.63; found: C 79.34, H 6.42, N 6.62.

3,4-Dibromo-1-methoxy-6-methyl-9*H*-carbazole (4)

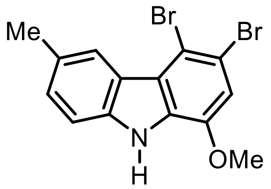

One drop of 48% aqueous HBr was added to a solution of 1-methoxy-6-methyl-9*H*-carbazole (**3**) (50.0 mg, 0.237 mmol) in dry acetonitrile (8 mL) at 0 °C and then, a solution of *N*-bromosuccinimide (86.5 mg, 0.486 mmol) in acetonitrile (7 mL) was added dropwise over a period of 10 min. Subsequently, the reaction mixture was stirred at room temperature for 30 min. After the reaction was finished, a saturated aqueous solution of sodium sulfite was added to the reaction mixture, the layers were separated, and the aqueous layer was extracted with dichloromethane (50 mL). The combined organic layers were washed with a saturated aqueous solution of sodium chloride and dried over magnesium sulfate. Removal of the solvent in vacuum and purification of the crude product by column chromatography on silica gel (petroleum ether/ethyl acetate, 9:1) afforded 3,4-dibromo-1-methoxy-6-methyl-9*H*-carbazole (**4**) as a light yellow solid; yield: 75.4 mg (0.204 mmol, 86%); mp: 198 °C.

UV (MeOH): λ = 232, 241 (sh), 248, 258, 265 (sh), 286, 295, 334, 349 nm.

Fluorescence (MeOH): λ_{ex} = 295 nm, λ_{em} = 370 nm.

IR (ATR): ν = 3434, 3083, 2963, 2928, 2852, 1560, 1447, 1431, 1392, 1275, 1253, 1111, 843, 806, 735, 681, 636 cm^{-1}.

^{1}H NMR (500 MHz, $CDCl_3$): δ = 2.53 (s, 3 H), 3.96 (s, 3 H), 7.09 (s, 1 H), 7.29 (dd, *J* = 8.3, 1.3 Hz, 1 H), 7.34 (d, *J* = 8.3 Hz, 1 H), 8.25 (br s, 1 H), 8.51 (s, 1 H).

^{13}C NMR and DEPT (125 MHz, $CDCl_3$): δ = 21.69 (CH_3), 56.00 (CH_3), 109.51 (C), 110.47 (CH), 110.54 (CH), 114.48 (C), 122.40 (CH), 123.26 (C), 123.28 (C), 128.11 (CH), 129.06 (C), 129.84 (C), 137.36 (C), 144.87 (C).

EI-MS: *m/z* (%) = 371 (48), 369 (100), 367 (54) [M^+], 356 (32), 354 (65), 352 (35), 324 (15), 244 (4), 164 (22), 138 (10).

Elemental analysis (%) calcd. for $C_{14}H_{11}Br_2NO$: C 45.56, H 3.00, N 3.80; found: C 45.66, H 3.03, N 3.81.

3,4-Dibromo-6-methyl-9*H*-carbazol-1-ol (5)

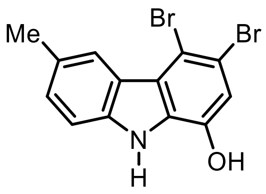

A 1 M solution of boron tribromide in dichloromethane (2.7 mL) was added to a solution of 3,4-dibromo-6-methyl-1-methoxy-9*H*-carbazole (**4**) (50.0 mg, 0.135 mmol) in 1,2-dichloroethane (20 mL). The reaction mixture was heated 84 °C for 30 min, then poured onto ice-water, and extracted with dichloromethane (3 × 20 mL). The organic layer was washed with water, then with a saturated aqueous solution of sodium chloride, and dried over magnesium sulfate. Removal of the solvent in vacuum and purification of the crude product by column chromatography on silica gel (petroleum ether/ethyl acetate, 4:1) provided 3,4-dibromo-6-methyl-9*H*-carbazol-1-ol (**5**) as a light brown solid; yield: 47.8 mg (0.134 mmol, 99%); mp: 157 °C (decomp.).

IR (ATR): v = 3426, 3228, 2915, 2850, 1440, 1285, 1265, 1241, 1181, 837, 802, 553 cm^{-1}.

^{1}H NMR (500 MHz, DMSO-d_6): δ = 3.35 (s, 3 H), 7.11 (s, 1 H), 7.29 (dd, *J* = 8.3, 1.1 Hz, 1 H), 7.43 (d, *J* = 8.3 Hz, 1 H), 8.37 (s, 1 H), 10.53 (s, 1 H), 11.54 (s, 1 H).

^{13}C NMR and DEPT (125 MHz, DMSO-d_6): δ = 21.44 (CH_3), 106.18 (C), 111.49 (CH), 112.82 (C), 113.53 (CH), 121.30 (CH), 122.24 (C), 122.53 (C), 127.44 (C), 127.88 (CH), 130.07 (C), 138.22 (C), 143.47 (C).

EI-MS: *m/z* (%) = 357 (47), 355 (100), 353 (49) [M^+], 274 (9), 246 (5), 195 (10), 166 (8), 137 (9).

HRMS (EI): *m/z* calcd. for $C_{13}H_9Br_2NO^+$ [M^+]: 352.9045; found: 352.9041.

Methyl 2-(phenylamino)benzoate

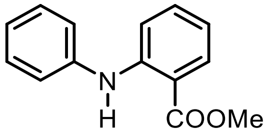

Aniline (**6**) (519 mg, 5.57 mmol) and then methyl 2-bromobenzoate (**7**) (1.00 g, 4.65 mmol) were added to a suspension of palladium(II) acetate (62.5 mg, 0.278 mmol), cesium carbonate (1.82 g, 5.59 mmol), and *rac*-BINAP (174 mg, 0.279 mmol) in dry toluene (40 mL). The reaction mixture was heated under reflux for 45 h. After cooling to room temperature, the reaction mixture was filtered with ethyl acetate (150 mL) through a short path of Celite and silica gel. Removal of the solvent in vacuum and purification of the crude product by column chromatography on silica gel (petroleum ether/ethyl acetate, 15:1) afforded methyl 2-(phenylamino)benzoate as a light yellow oil; yield: 867 mg (3.82 mmol, 82%).

^{1}H NMR (500 MHz, $CDCl_3$): δ = 3.90 (s, 3 H), 6.73 (ddd, *J* = 8.1, 7.0, 1.2 Hz, 1 H), 7.09 (ddd, *J* = 8.4, 2.1, 1.1 Hz, 1 H), 7.23–7.26 (m, 3 H), 7.29–7.36 (m, 3 H), 7.96 (dd, *J* = 8.0, 1.6 Hz, 1 H), 9.46 (br s, 1 H).

^{13}C NMR and DEPT (125 MHz, $CDCl_3$): δ = 51.76 (CH_3), 111.84 (C), 113.98 (CH), 117.06 (CH), 122.48 (2 CH), 123.52 (CH), 129.33 (2 CH), 131.59 (CH), 134.07 (CH), 140.71 (C), 147.90 (C), 168.91 (C=O).

For further spectroscopic data, see ref.[9].

Methyl 9*H*-carbazol-1-carboxylate (8)

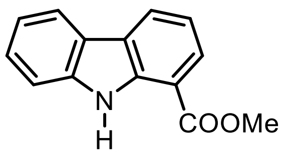

Palladium(II) acetate (410 mg, 1.83 mmol) was added to a solution of methyl 2-(phenylamino)benzoate (277 mg, 1.22 mmol) in acetic acid (15 mL) and the reaction mixture was heated under reflux for 2 h. After cooling to room temperature, the mixture was filtered with dichloromethane (150 mL) through a short path of Celite and silica gel. Removal of the solvent in vacuum and purification of the crude product by column chromatography on silica gel (petroleum ether/ethyl acetate, 15:1) provided methyl 9*H*-carbazol-1-carboxylate (**8**) as a light yellow solid; yield: 176 mg (0.781 mmol, 64%); mp: 136–138 °C (ref.[10]: 135–137 °C).

UV (MeOH): λ = 241, 248, 278, 302, 358 nm.

IR (ATR): ν = 3406, 3057, 2949, 2849, 1677, 1600, 1492, 1457, 1434, 1300, 1260, 1218, 1206, 1137, 1068, 750, 721, 679, 621, 558 cm^{-1}.

^{1}H NMR (500 MHz, $CDCl_3$): δ = 4.02 (s, 3 H), 7.23–7.29 (m, 2 H), 7.45–7.48 (m, 1 H), 7.52 (d, *J* = 8.1 Hz, 1 H), 8.06–8.10 (m, 2 H), 8.27 (d, *J* = 7.6 Hz, 1 H), 9.92 (br s, 1 H).

^{13}C NMR and DEPT (125 MHz, $CDCl_3$): δ = 51.99 (CH_3), 111.08 (CH), 111.57 (C), 118.46 (CH), 119.94 (CH), 120.39 (CH), 122.41 (C), 124.61 (C), 125.48 (CH), 126.53 (CH), 127.34 (CH), 139.61 (C), 140.02 (C), 167.89 (C=O).

EI-MS: *m/z* (%) = 225 (59) [M^+], 193 (100), 165 (51), 139 (20).

Elemental analysis (%) calcd. for $C_{14}H_{11}NO_2$: C 74.65, H 4.92, N 6.22; found: C 74.25, H 5.10, N 6.15.

For further spectroscopic data, see ref.[11].

Methyl 3,6-dibromo-9*H*-carbazol-1-carboxylate (9)

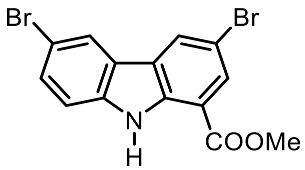

Freshly recrystallized pyridinium tribromide (354 mg, 1.11 mmol) was added to a solution of methyl 9*H*-carbazol-1-carboxylate (**8**) (50.0 mg, 0.222 mmol) in dry ethanol (20 mL) and the mixture was stirred at room temperature for 6 h. After addition of a saturated aqueous solution of sodium sulfite (30 mL), the layers were separated, and the aqueous layer was extracted with dichloromethane (3 × 30 mL). The combined organic layers were washed with a saturated aqueous solution of sodium chloride and dried over magnesium sulfate. Removal of the solvent in vacuum and purification of the crude product by column chromatography on silica gel (petroleum ether/ethyl acetate, 9:1) afforded methyl 3,6-dibromo-9*H*-carbazol-1-carboxylate (**9**) as a light yellow solid; yield: 83.0 mg (0.217 mmol, 98%); mp: 205–206 °C.

UV (MeOH): λ = 223, 246, 287, 315, 374 nm.

Fluorescence (MeOH): λ_{ex} = 287 nm, λ_{em} = 418 nm.

IR (ATR): ν = 3396, 3095, 2953, 2919, 2850, 1674, 1472, 1434, 1270, 1244, 1219, 1203, 1173, 1077, 864, 804, 786, 726, 641 cm^{-1}.

^{1}H NMR (500 MHz, $CDCl_3$): δ = 4.02 (s, 3 H), 7.39 (d, *J* = 8.6 Hz, 1 H), 7.57 (dd, *J* = 8.6, 1.9 Hz, 1 H), 8.15 (d, *J* = 1.9 Hz, 1 H), 8.18 (d, *J* = 1.9 Hz, 1 H), 8.30 (d, *J* = 1.8 Hz, 1 H), 9.92 (br s, 1 H).

^{13}C NMR and DEPT (125 MHz, $CDCl_3$): δ = 52.39 (CH_3), 111.30 (C), 112.75 (CH), 113.13 (C), 113.17 (C), 123.16 (C), 123.42 (CH), 125.28 (C), 128.21 (CH), 130.09 (CH), 130.35 (CH), 138.53 (C), 138.93 (C), 166.62 (C=O).

EI-MS (200 °C): *m/z* (%) = 385 (47), 383 (100), 381 (47) [M^+], 353 (41), 351 (87), 349 (41), 322 (3), 303 (7), 243 (14), 164 (19), 122 (11).

HRMS (EI): *m/z* calcd. for $C_{14}H_9Br_2NO_2^+$ [M^+]: 380.8995; found: 380.9002.

3,6-Dibromo-9*H*-carbazol-1-carboxylic acid (10)

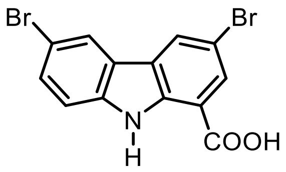

A solution of potassium hydroxide (73.3 mg, 1.31 mmol) in water (8 mL) was added to a solution of methyl 3,6-dibromo-9*H*-carbazol-1-carboxylate (**9**) (30.2 mg, 0.079 mmol) in THF (16 mL) and the mixture was stirred at room temperature for 24 h. After addition of dichloromethane (15 mL), the mixture was neutralized with 2 N aqueous HCl and the aqueous layer was extracted with dichloromethane (3 × 15 mL). The combined organic layers were washed with a saturated aqueous solution of sodium chloride and dried over magnesium sulfate. Removal of the solvent in vacuum and purification of the crude product by column chromatography on silica gel (petroleum ether/ethyl acetate, 5:1 + ethyl acetate/1% HOAc) provided 3,6-dibromo-9*H*-carbazol-1-carboxylic acid (**10**) as a light yellow solid; yield: 29.1 mg (0.079 mmol, 100%); mp: 260 °C (decomp.).

UV (MeOH): λ = 226, 244 (sh), 254 (sh), 280, 311, 351, 365 nm.

Fluorescence (MeOH): λ_{ex} = 351 nm, λ_{em} = 524 nm.

IR (ATR): ν = 3429, 3404, 2835, 2531, 1682, 1426, 1272, 1240, 1216, 1186, 920, 873, 799, 788, 704, 671, 643, 611 cm^{-1}.

^{1}H NMR (500 MHz, DMSO-d_6): δ = 7.59 (dd, *J* = 8.6, 1.9 Hz, 1 H), 7.69 (d, *J* = 8.6 Hz, 1 H), 8.06 (d, *J* = 1.9 Hz, 1 H), 8.50 (d, *J* = 1.7 Hz, 1 H), 8.74 (d, *J* = 1.7 Hz, 1 H), 11.65 (s, 1 H), 13.60 (br s, 1 H).

^{13}C NMR and DEPT (125 MHz, DMSO-d_6): δ = 109.97 (C), 111.87 (C), 114.43 (CH), 114.66 (C), 122.64 (C), 123.41 (CH), 125.28 (C), 128.24 (CH), 129.46 (CH), 129.92 (CH), 137.98 (C), 139.44 (C), 166.49 (C=O).

EI-MS: *m/z* (%) = 371 (36), 369 (77), 367 (37) [M^+], 353 (46), 351 (100), 349 (47), 321 (10), 242 (34), 163 (21), 121 (12).

HRMS (EI): *m/z* calcd. for $C_{13}H_7Br_2NO_2^+$ [M^+]: 366.8838; found: 366.8827.

1-Hydroxymethyl-9*H*-carbazole (11)

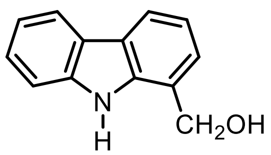

A 1 M solution of diisobutylaluminum hydride in dichloromethane (1.11 mmol) was added dropwise to a solution of methyl 9*H*-carbazol-1-carboxylate (**8**) (100 mg, 0.444 mmol) in dichloromethane (10 mL) at –78 °C. The reaction mixture was stirred at –78 °C for 30 min, warmed to room temperature, and stirred for additional 30 min at room temperature. After addition of water (10 mL), the layers were separated, and the aqueous layer was extracted with dichloromethane (3 × 10 mL). The combined organic layers were washed with a saturated aqueous solution of sodium chloride and dried over magnesium sulfate. Removal of the solvent in vacuum and purification of the crude product by column chromatography on silica gel (petroleum ether/ethyl acetate, 4:1) afforded 1-hydroxymethyl-9*H*-carbazole (**11**) as a light yellow solid; yield: 84.6 mg (0.429 mmol, 97%); mp: 128–130 °C (ref.[3]: 136–137 °C).

UV (MeOH): λ = 228 (sh), 235, 247 (sh), 258, 282 (sh), 292, 323, 335 nm.

IR (ATR): ν = 3521, 3337, 3054, 2919, 2853, 1455, 1433, 1335, 1235, 1006, 967, 738, 711, 648, 617, 562, 528 cm^{-1}.

^{1}H NMR (500 MHz, $CDCl_3$): δ = 1.97 (br s, 1 H), 5.07 (s, 2 H), 7.17 (t, *J* = 7.6 Hz, 1 H), 7.22–7.24 (m, 2 H), 7.40–7.46 (m, 2 H), 8.03 (d, *J* = 7.6 Hz, 1 H), 8.08 (d, *J* = 7.6 Hz, 1 H), 8.86 (br s, 1 H).

^{13}C NMR and DEPT (125 MHz, $CDCl_3$): δ = 64.54 (CH_2), 110.83 (CH), 118.90 (CH), 119.37 (CH), 120.08 (CH), 120.31 (CH), 122.49 (C), 123.03 (C), 123.74 (C), 123.85 (CH), 125.90 (CH), 138.40 (C), 139.55 (C).

EI-MS: *m/z* (%) = 197 (48) [M^+], 179 (100), 167 (17), 152 (19).

Elemental analysis (%) calcd. for $C_{13}H_{11}NO$: C 79.16, H 5.62, N 7.10; found: C 79.36, H 5.84, N 6.87.

3,6-Dibromo-1-hydroxymethyl-9*H*-carbazole (Adhibin) (12)

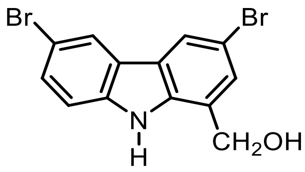

Freshly recrystallized pyridinium tribromide (97.0 mg, 0.303 mmol) was added to a solution of 1-hydroxymethyl-9*H*-carbazole (**11**) (20.0 mg, 0.101 mmol) in dry ethanol (15 mL) and the mixture was stirred at room temperature for 5 h. After addition of a saturated aqueous solution of sodium sulfite (30 mL), the layers were separated, and the aqueous layer was extracted with dichloromethane (3 × 30 mL). The combined organic layers were washed with a saturated aqueous solution of sodium chloride and dried over magnesium sulfate. Removal of the solvent in vacuum and purification of the crude product by column chromatography on silica gel (petroleum ether/ethyl acetate, 4:1) provided 3,6-dibromo-1-hydroxymethyl-9*H*-carbazole (adhibin) (**12**) as a light yellow solid; yield: 33.0 mg (0.093 mmol, 92%); mp: 161–162 °C.

UV (MeOH): λ = 241, 250, 268, 302, 337, 351 nm.

Fluorescence (MeOH): λ_{ex} = 268 nm, λ_{em} = 374 nm.

IR (ATR): ν = 3547, 3241, 2951, 2921, 1716, 1603, 1478, 1295, 1225, 988, 968, 852, 791, 660, 636 cm^{-1}.

^{1}H NMR (500 MHz, $CDCl_3$): δ = 2.02 (br s, 1 H), 5.05 (s, 2 H), 7.32 (d, *J* = 8.6 Hz, 1 H), 7.34 (d, *J* = 1.7 Hz, 1 H), 7.51 (dd, *J* = 8.6, 1.9 Hz, 1 H), 8.05 (d, *J* = 1.7 Hz, 1 H), 8.11 (d, *J* = 1.9 Hz, 1 H), 8.93 (br s, 1 H).

^{13}C NMR and DEPT (125 MHz, $CDCl_3$): δ = 63.96 (CH_2), 111.90 (C), 112.48 (CH), 112.53 (C), 122.65 (CH), 123.24 (CH), 123.78 (C), 124.38 (C), 124.42 (C), 126.86 (CH), 129.35 (CH), 137.41 (C), 138.36 (C).

EI-MS: *m/z* (%) = 357 (31), 355 (71), 353 (40) [M^+], 339 (46), 337 (100), 335 (24), 256 (25), 177 (23), 150 (7), 128 (7).

HRMS (EI): *m/z* calcd. for $C_{13}H_9Br_2NO^+$ [M^+]: 352.9045; found: 352.9032.

NMR SPECTRA

2-Methoxy-*N*-(*p*-tolyl)aniline

^{1}H NMR (500 MHz, $CDCl_3$)

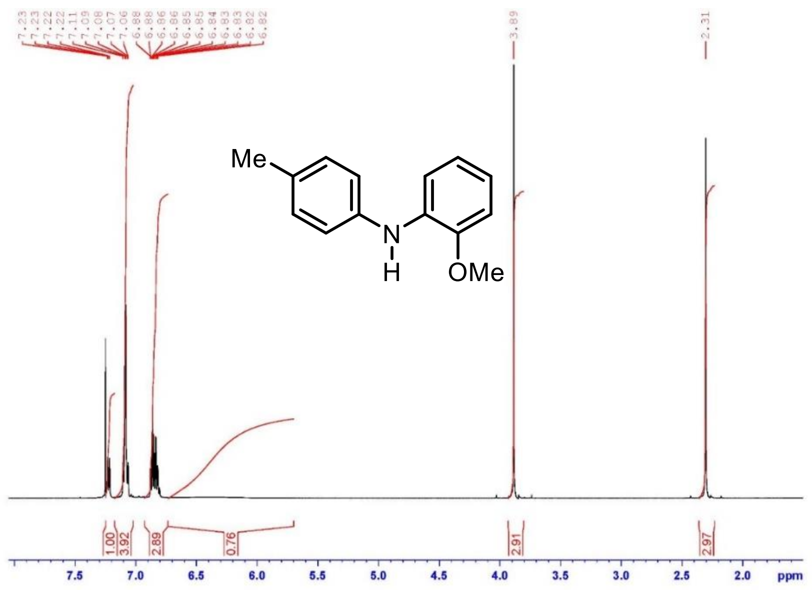

2-Methoxy-*N*-(*p*-tolyl)aniline

^{13}C NMR (125 MHz, $CDCl_3$)

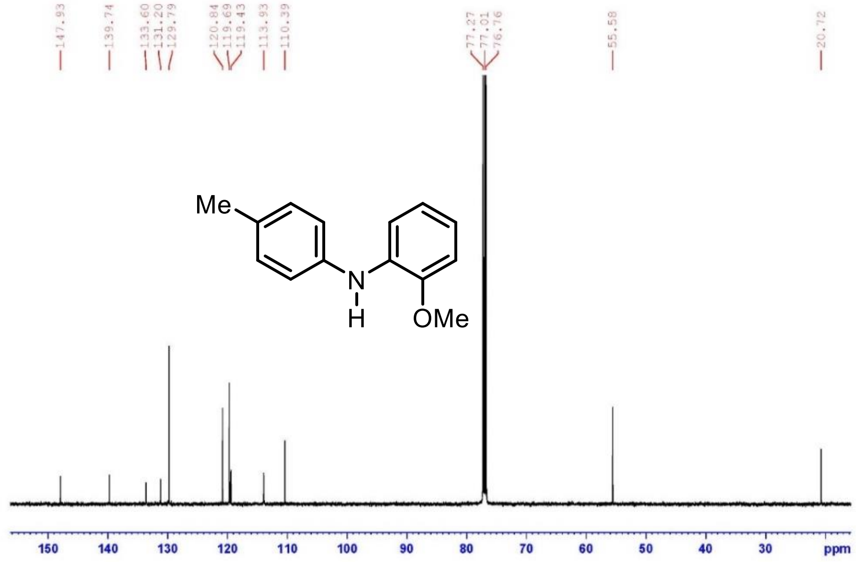

1-Methoxy-6-methyl-9*H*-carbazole (**3**)

^{1}H NMR (500 MHz, $CDCl_3$)

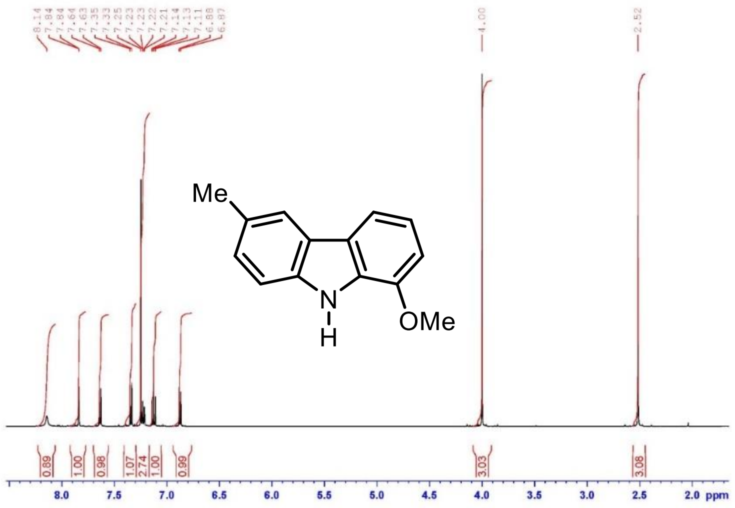

1-Methoxy-6-methyl-9*H*-carbazole (**3**)

^{13}C NMR (125 MHz, $CDCl_3$)

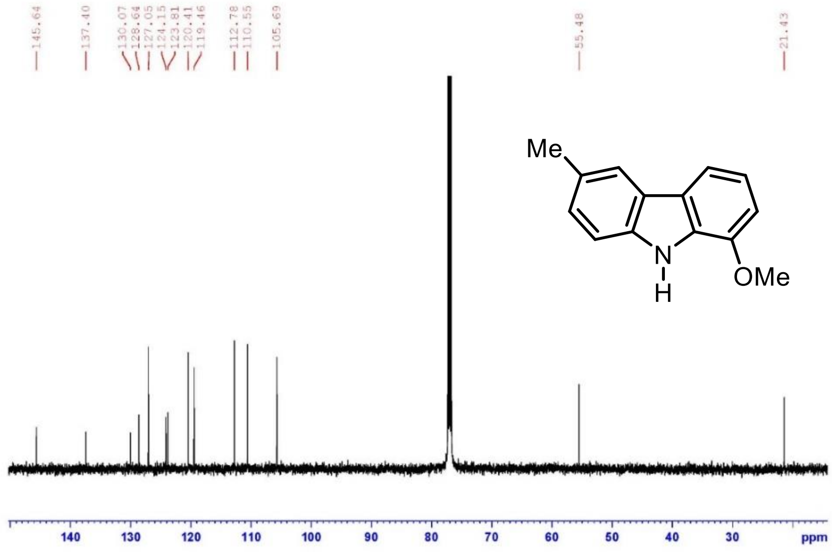

3,4-Dibromo-1-methoxy-6-methyl-9*H*-carbazole (**4**)

^{1}H NMR (500 MHz, $CDCl_3$)

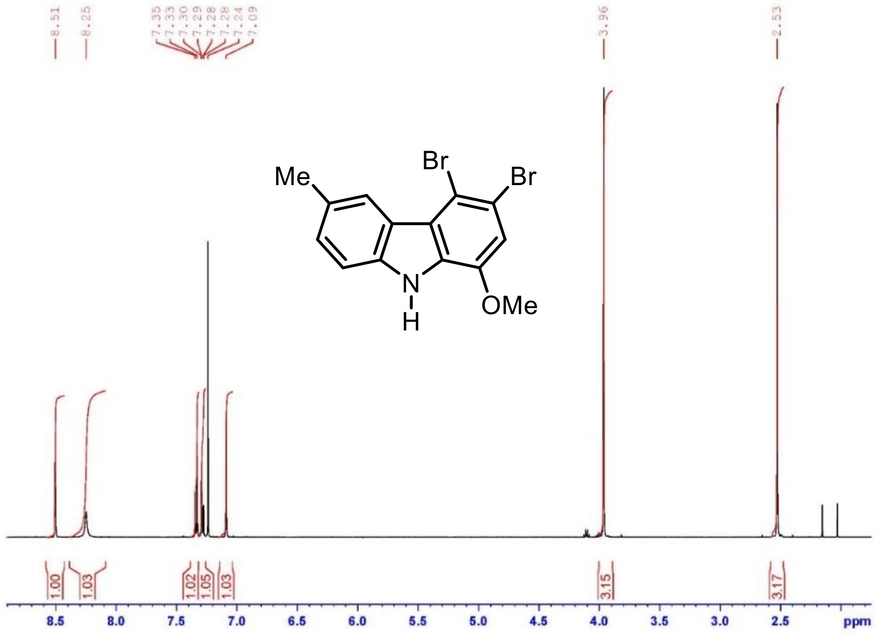

3,4-Dibromo-1-methoxy-6-methyl-9*H*-carbazole (**4**)

^{13}C NMR (125 MHz, $CDCl_3$)

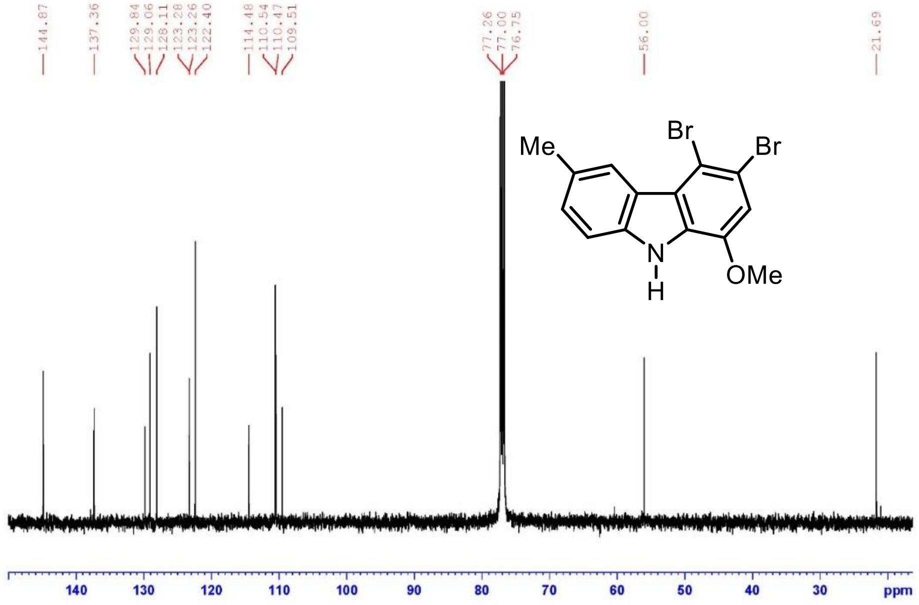

3,4-Dibromo-6-methyl-9*H*-carbazol-1-ol (**5**)

^{1}H NMR (500 MHz, DMSO-d$_6$)

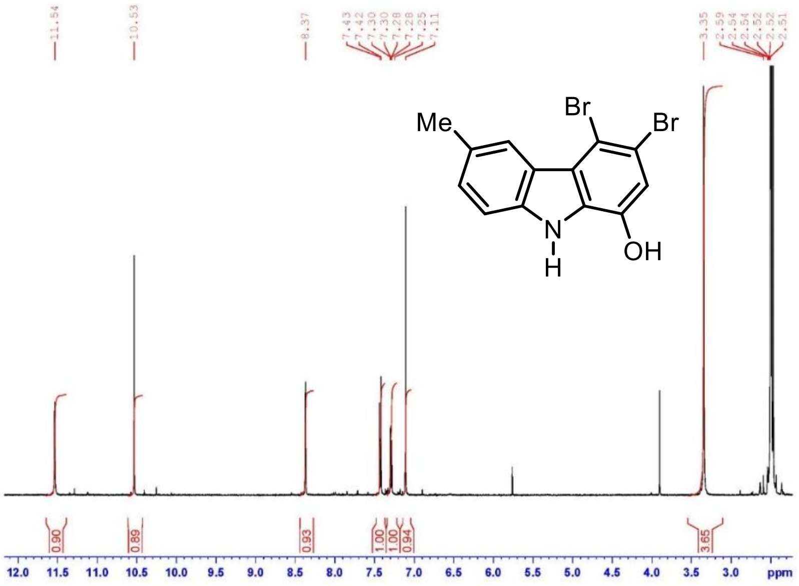

3,4-Dibromo-6-methyl-9*H*-carbazol-1-ol (**5**)

^{13}C NMR (125 MHz, DMSO-d$_6$)

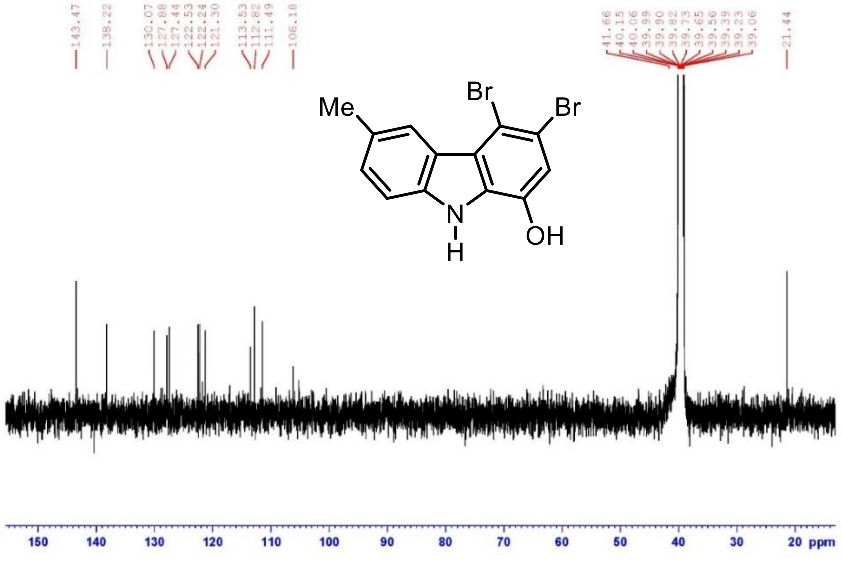

Methyl 2-(phenylamino)benzoate

^{1}H NMR (500 MHz, $CDCl_3$)

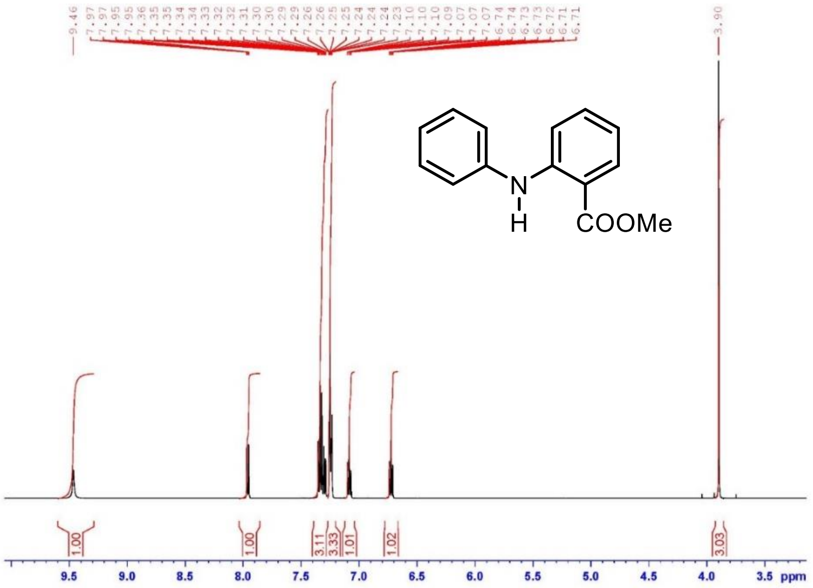

Methyl 2-(phenylamino)benzoate

^{13}C NMR (125 MHz, $CDCl_3$)

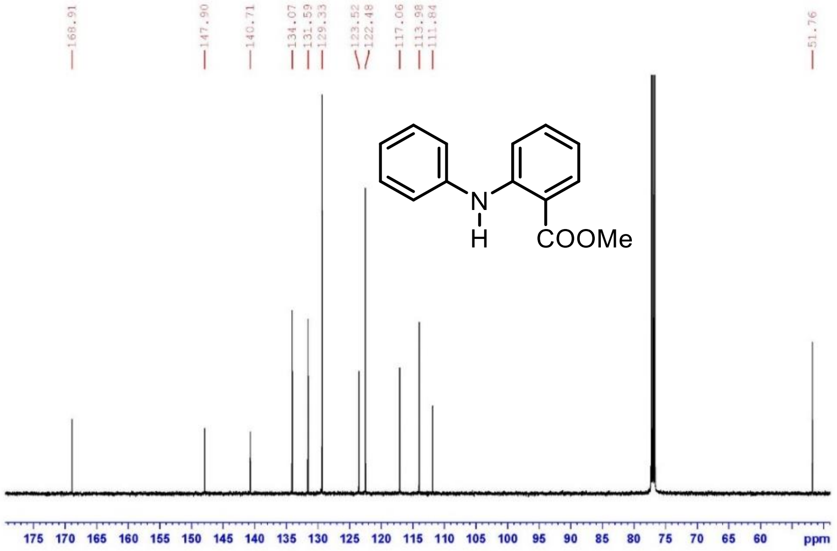

Methyl 9*H*-carbazol-1-carboxylate (**8**)

^{1}H NMR (500 MHz, $CDCl_3$)

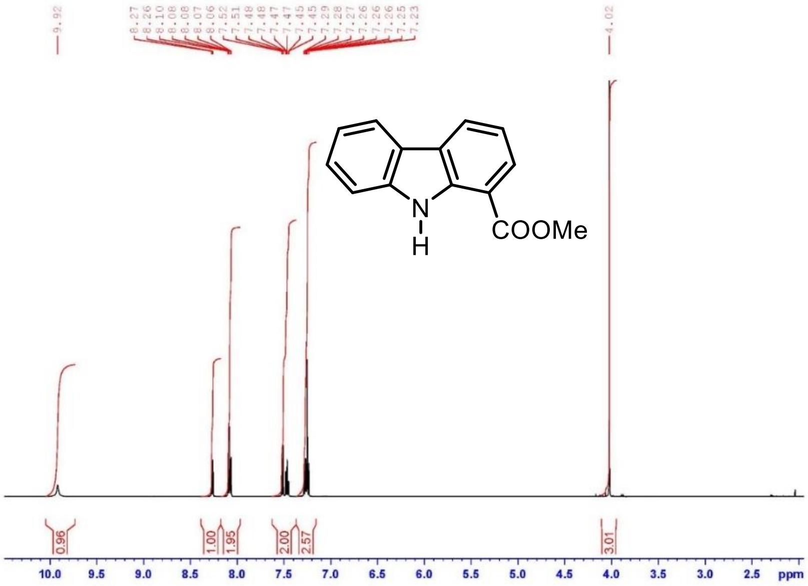

Methyl 9*H*-carbazol-1-carboxylate (**8**)

^{13}C NMR (125 MHz, $CDCl_3$)

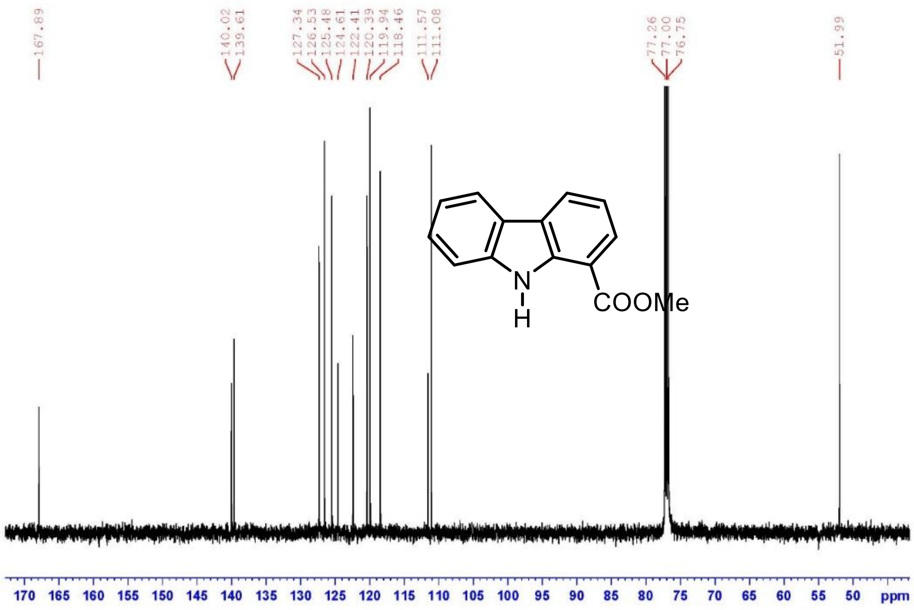

Methyl 3,6-dibromo-9*H*-carbazol-1-carboxylate (**9**)

^{1}H NMR (500 MHz, $CDCl_3$)

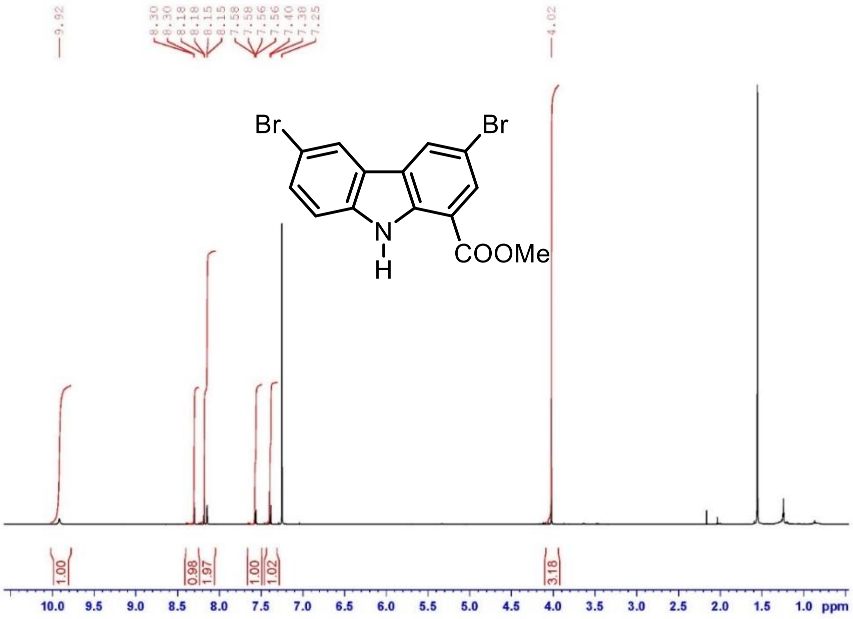

Methyl 3,6-dibromo-9*H*-carbazol-1-carboxylate (**9**)

^{13}C NMR (125 MHz, $CDCl_3$)

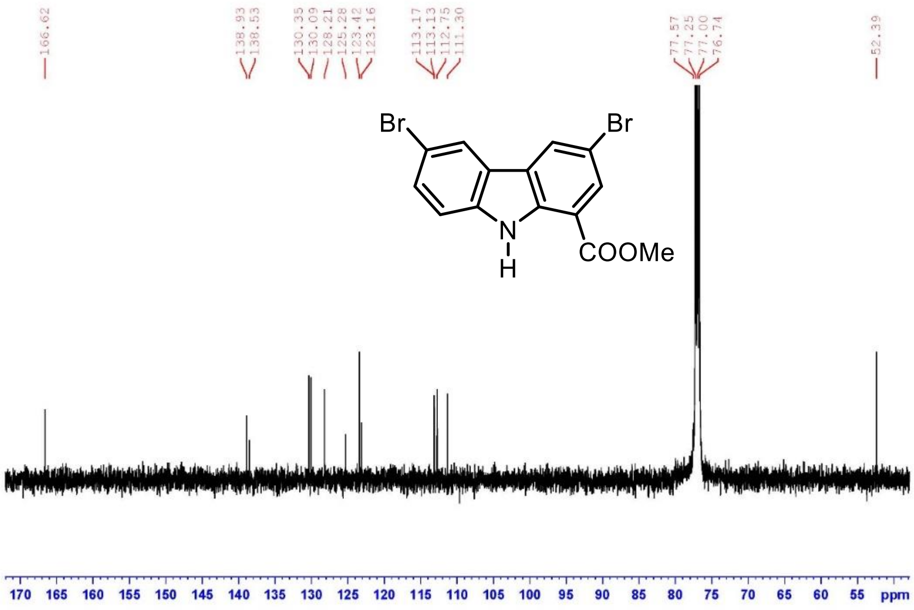

3,6-Dibromo-9*H*-carbazol-1-carboxylic acid (**10**)

^{1}H NMR (500 MHz, DMSO-d_6)

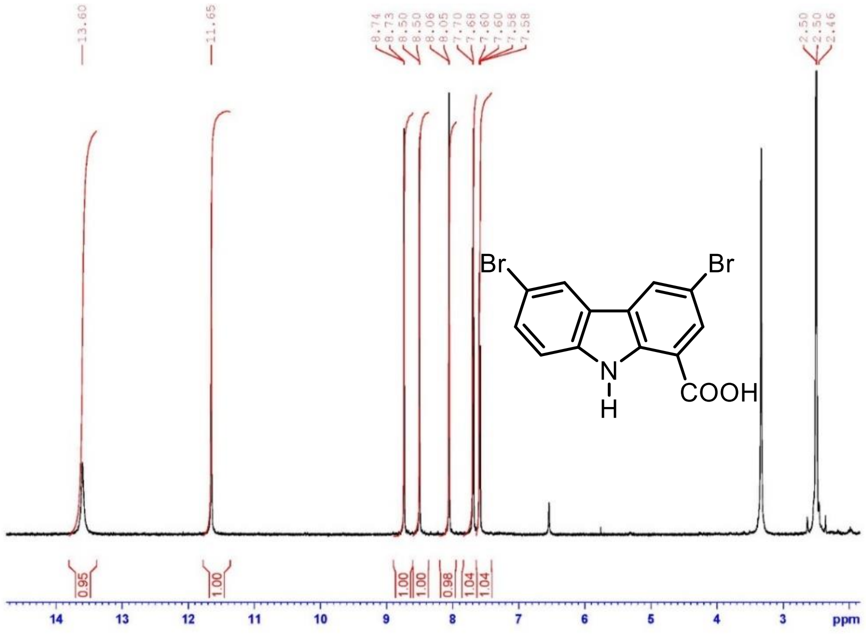

3,6-Dibromo-9*H*-carbazol-1-carboxylic acid (**10**)

^{13}C NMR (125 MHz, DMSO-d_6)

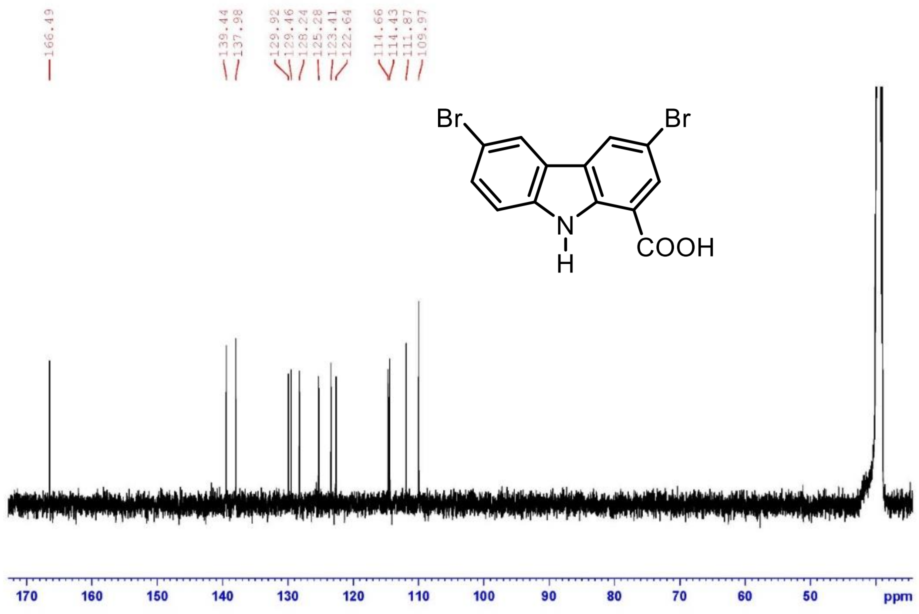

1-Hydroxymethyl-9*H*-carbazole (**11**)

^{1}H NMR (500 MHz, $CDCl_3$)

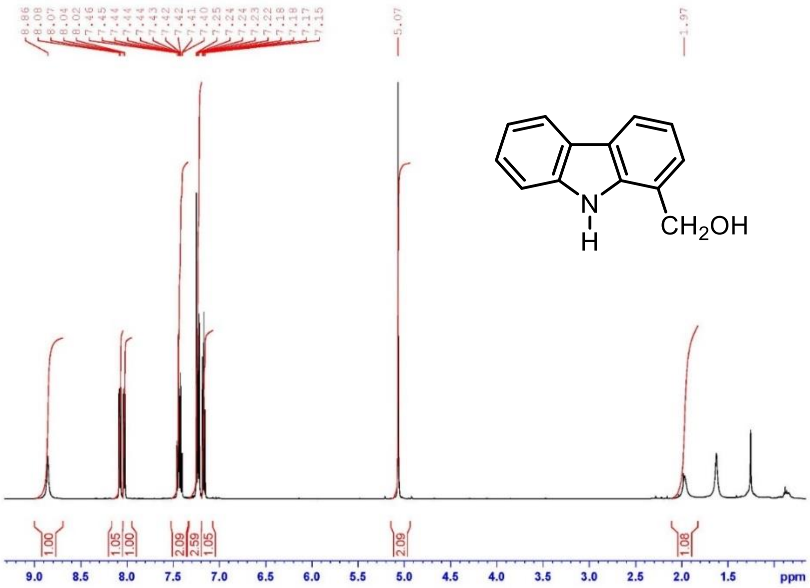

1-Hydroxymethyl-9*H*-carbazole (**11**)

^{13}C NMR (125 MHz, $CDCl_3$)

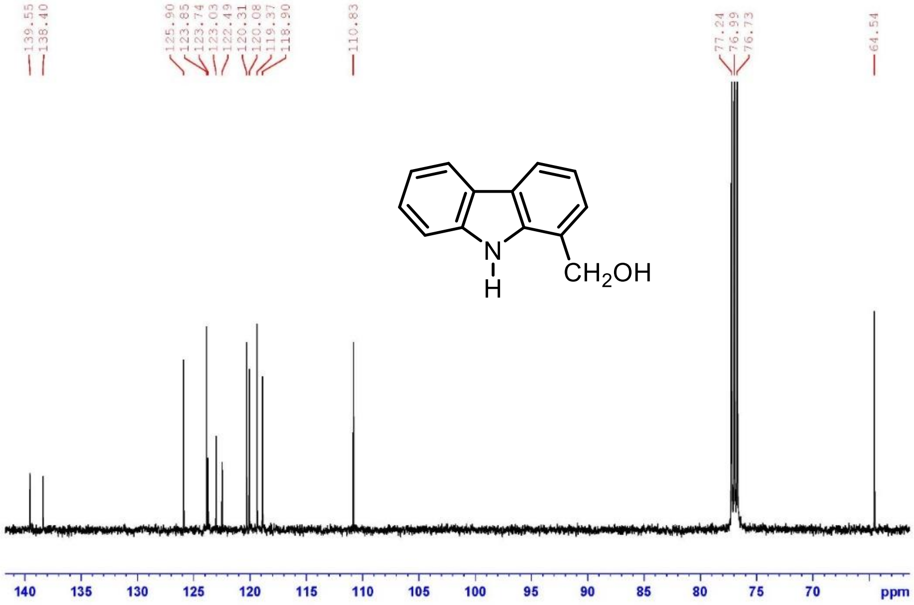

3,6-Dibromo-1-hydroxymethyl-9*H*-carbazole (Adhibin) (**12**)

^{1}H NMR (500 MHz, $CDCl_3$)

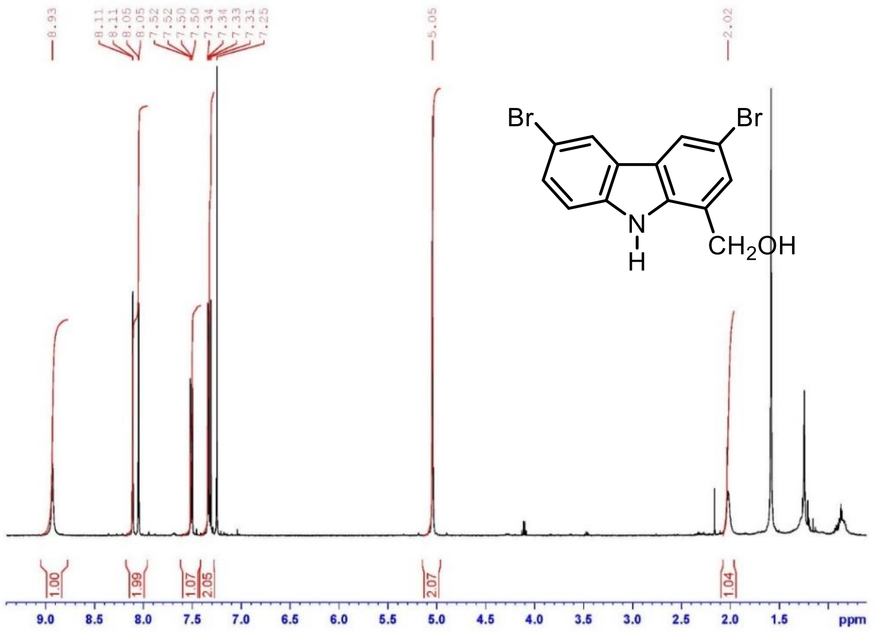

3,6-Dibromo-1-hydroxymethyl-9*H*-carbazole (Adhibin) (**12**)

^{13}C NMR (125 MHz, $CDCl_3$)

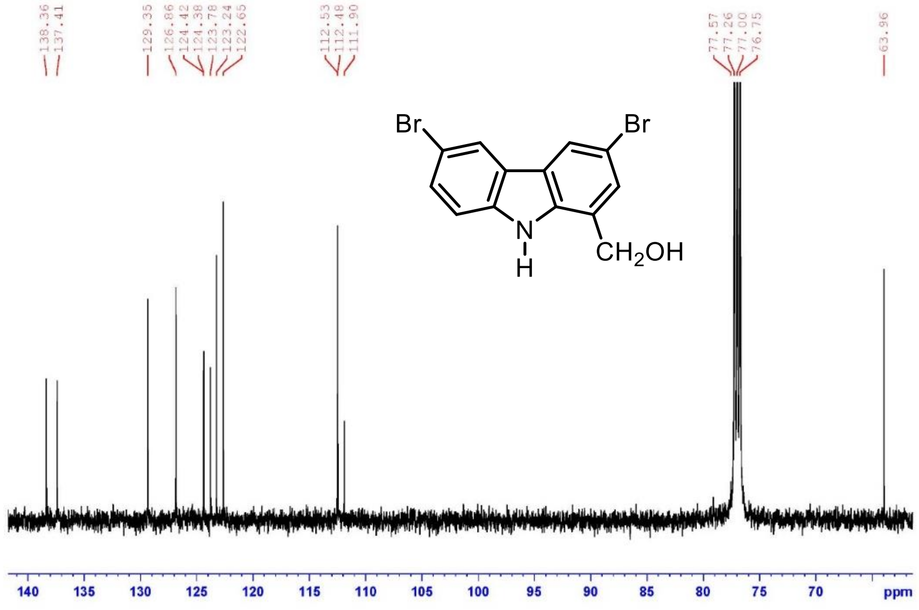

SUPPLEMENTARY REFERENCES

1. Amrute-Nayak, M. *et al.* Transformation of the Nonprocessive Fast Skeletal Myosin II into a Processive Motor. *Small* **15**, (2019).

2. Deacon, J. C., Bloemink, M. J., Rezavandi, H., Geeves, M. A. & Leinwand, L. A. Identification of functional differences between recombinant human α and β cardiac myosin motors. *Cellular and Molecular Life Sciences* **69**, 2261–2277 (2012).

3. Fujita-Becker, S. *et al.* Functional Characterization of the N-terminal Region of Myosin-2. *Journal of Biological Chemistry* **281**, 36102–36109 (2006).

4. Taft, M. H. *et al.* Dictyostelium myosin-5b is a conditional processive motor. *Journal of Biological Chemistry* **283**, 26902–26910 (2008).

5. Nalavadi, V. *et al.* Kinetic Mechanism of Myosin IXB and the Contributions of Two Class IX-specific Regions. *Journal of Biological Chemistry* **280**, 38957–38968 (2005).

6. Liao, W., Elfrink, K. & Bähler, M. Head of Myosin IX Binds Calmodulin and Moves Processively toward the Plus-end of Actin Filaments. *Journal of Biological Chemistry* **285**, 24933–24942 (2010).

7. Diensthuber, R. P. *et al.* Phalloidin perturbs the interaction of human non-muscle myosin isoforms 2A and 2C1 with F-actin. *FEBS Lett* **585**, 767–771 (2011).

8. Pathan-Chhatbar, S. *et al.* Three mammalian tropomyosin isoforms have different regulatory effects on nonmuscle myosin-2B and filamentous β-actin in vitro. *Journal of Biological Chemistry* **293**, 863–875 (2018).

9. Rao, H., Jin, Y., Fu, H., Jiang, Y. & Zhao, Y. A versatile and efficient ligand for copper-catalyzed formation of C-N, C-O, and P-C bonds: Pyrrolidine-2-phosphonic acid phenyl monoester. *Chemistry - A European Journal* **12**, 3636–3646 (2006).

10. Julia, M.; Lenzi, J. *Preparation d'acides Tetrahydro-1,2,3,4-Carbazole-1 Ou -4.* vols 2263–1267 (Bull.Soc.Chim.France, 1962).

11. Molina, P., Fresneda, P. M. & Almendros, P. Fused carbazoles by tandem Aza Wittig/electrocyclic ring closure. Preparation of 6H-pyrido[4,3-b] carbazole, 11H-pyrido[4,3-a]carbazole and 11H-pyrido[3,4-a]carbazole derivatives. *Tetrahedron* **49**, 1223–1236 (1993).
