# Supplementary material for: An allosteric inhibitor of RhoGAP class-IX myosins suppresses the metastatic features of cancer cells
Source: Nat Commun. 2024 Nov 16;15:9947. doi: 10.1038/s41467-024-54181-6 (PMC11569205; doi:10.1038/s41467-024-54181-6)
Supplement: Supplementary file 2 — Description of Additional Supplementary Files [file 41467_2024_54181_MOESM2_ESM.pdf]

An allosteric inhibitor of RhoGAP class-IX myosins suppresses the metastatic features of cancer cells

Despoina Kyriazi[1], Lea Voth[1], Almke Bader[1], Wiebke Ewert[1,2], Juliane Gerlach[3], Kerstin Elfrink[4], Peter Franz[1], Mariana I. Tsap[5], Bastian Schirmer[6], Julia Damiano-Guercio[1], Falk K. Hartmann[1], Masina Plenge[7], Azam Salari[8], Dennis Schoettelndreier[1], Katharina Strienke[1], Nadine Bresch[1], Claudio Salinas[1], Herwig O. Gutzeit[9,§], Nora Schaumann[10], Kais Hussein[11], Heike Bähre[12], Inga Brüsch[13], Peter Claus[14], Detlef Neumann[6,§], Manuel H. Taft[1§], Halyna R. Shcherbata[5,§], Anaclet Ngezahayo[7,§], Martin Bähler[4,§], Mahdi Amiri[8,§], Hans-Joachim Knölker[3,§], Matthias Preller[1,2,§], and Georgios Tsiavaliaris[1,15,§,*]

DESCRIPTION TO SUPPLEMENTARY DATA

- Supplementary movie legends
- Description of Supplementary Data 1

SUPPLEMENTARY MOVIE LEGENDS

Supplementary Movie 1. Adhibin causes the detachment of A459 and MLE-12 cells. A549 and MLE-12 cells treated with 1% DMSO or 25 µM adhibin and imaged on glass-bottom dishes using brightfield microscopy and a 60x objective. Time is indicated in hr:min. Scale bar: 20 µm.

Supplementary Movie 2. Adhibin impairs B16-F1 spreading. Untreated control B16-F1 cells and 1% DMSO or 5 µM adhibin treated B16-F1 cells, were imaged in phase contrast to monitor spreading on laminin-coated glass-bottom dishes using a 10× objective. Time is indicated in min:sec. Scale bar is 100 µm.

Supplementary Movie 3. Adhibin impairs B16-F1 random migration. Untreated control B16-F1 cells and 1% DMSO, 5 µM, or 10 µM adhibin treated B16-F1 cells, were imaged in DIC to monitor random migration on laminin-coated glass-bottom dishes using a 10× objective. Time is indicated in hr:min. Scale bar is 100 µm.

Supplementary Movie 4. Adhibin impairs B16-F1 lamellipodia architecture and dynamics. 1% DMSO or 5 µM adhibin treated B16-F1 cells, were imaged with bright field microscopy to closely resolve the lammellipodium on laminin-coated glass-bottom dishes using a 60x objective. B16-F1 cells were also transfected with mscarlet-LifeAct, treated with 1% DMSO or 5 µM adhibin and imaged using TIRF-microscopy on laminin-coated glass-bottom dishes using a 60x objective. For FRAP experiments, B16-F1 cells were transfected with EGPF-β-actin, treated with 1% DMSO or 5 µM adhibin and imaged using confocal microscopy on laminin-coated glass-bottom dishes using a 63× objective. The area was bleached for 0.5ms. Time is indicated in min:sec. Scale bar is 5 µm. Time is indicated in min:sec. Scale bar: 20 µm.

Supplementary Movie 5. Adhibin affects Rho localization in B16-F1 cells. B16-F1 cells were transfected with dtomato-rhotekin, treated with 1% DMSO or 5 µM adhibin and imaged using TIRF-microscopy on laminin-coated glass-bottom dishes using a 60x objective. Time is indicated in min:sec. Scale bar: 20 µm.

Supplementary Movie 6. Adhibin impairs cell division. B16-F1 cells were treated with 1% DMSO or 5 µM adhibin and imaged on laminin-coated glass-bottom dishes with DIC microscopy using a 10x objective and with brightfield microscopy using a 60x objective. Time is indicated in hours:min:sec. Scale bar: 20 µm.

DESCRIPTION OF SUPPLEMENTARY DATA 1 HOMOLOGY MODELS

Homology Model 1. Homology model of *Rn*Myo9a.

Homology Model 2. Homology model of *Rn*Myo9a in complex with adhibin.

Homology Model 3. Homology model of *Rn*Myo9b.

Homology Model 4. Homology model of *Rn*Myo9b in complex with adhibin.
