# Supplementary material for: An allosteric inhibitor of RhoGAP class-IX myosins suppresses the metastatic features of cancer cells
Source: Nat Commun. 2024 Nov 16;15:9947. doi: 10.1038/s41467-024-54181-6 (PMC11569205; doi:10.1038/s41467-024-54181-6)
Supplement: Supplementary file 10 — Reporting Summary [file 41467_2024_54181_MOESM10_ESM.pdf]

Corresponding author(s): Georgios Tsiavaliaris

Last updated by author(s): Oct 24, 2024

Reporting Summary

Nature Portfolio wishes to improve the reproducibility of the work that we publish. This form provides structure for consistency and transparency in reporting. For further information on Nature Portfolio policies, see our Editorial Policies and the Editorial Policy Checklist.

Statistics

For all statistical analyses, confirm that the following items are present in the figure legend, table legend, main text, or Methods section.

n/a	Confirmed	
☐	☒	The exact sample size (*n*) for each experimental group/condition, given as a discrete number and unit of measurement
☐	☒	A statement on whether measurements were taken from distinct samples or whether the same sample was measured repeatedly
☐	☒	The statistical test(s) used AND whether they are one- or two-sided<br>*Only common tests should be described solely by name; describe more complex techniques in the Methods section.*
☒	☐	A description of all covariates tested
☐	☒	A description of any assumptions or corrections, such as tests of normality and adjustment for multiple comparisons
☐	☒	A full description of the statistical parameters including central tendency (e.g. means) or other basic estimates (e.g. regression coefficient) AND variation (e.g. standard deviation) or associated estimates of uncertainty (e.g. confidence intervals)
☐	☒	For null hypothesis testing, the test statistic (e.g. *F*, *t*, *r*) with confidence intervals, effect sizes, degrees of freedom and *P* value noted<br>*Give P values as exact values whenever suitable.*
☒	☐	For Bayesian analysis, information on the choice of priors and Markov chain Monte Carlo settings
☒	☐	For hierarchical and complex designs, identification of the appropriate level for tests and full reporting of outcomes
☐	☒	Estimates of effect sizes (e.g. Cohen's *d*, Pearson's *r*), indicating how they were calculated

Our web collection on statistics for biologists contains articles on many of the points above.

Software and code

Policy information about availability of computer code

Data collection

Diffraction data was collected at Soleil synchrotron (France) beamline Proxima-2A.

Microscale Thermophoresis:
Monolith NT.115 Microscale Thermophoresis device

Spectroscopic measurements:
BioTek platereader

Trans epithelial Electrical Resistance (TEER) Assay:
cellZscope (nanoAnalytics, Muenster, Germany)

ÄKTA pure™ Chromatographysystem
(GE Healthcare)

StepOnePlus Real-Time PCR system (Applied Biosystems, Thermo Fisher Scientific)

Microscopes:
ZEISS 980 with Airyscan 2
Olympus IX-83 TIRF inverted microscope

Olympus IX-81 TIRF inverted microscope
Eclipse TS1000 brightfield microscope (Nikon)
Nikon microcope with Andor/Yokogava Spinning disk
Zeiss LSM 700

Data analysis

Fiji, Schindelin et al., https://hpc.nih.gov/apps/Fiji
Excel, Microsoft, https://www.microsoft.com/en-us/microsoft-365/excel
XDS, XDS Program Package, https://xds.mr.mpg.de/
AIMLESS, Ccp4 software suite, https://www.mrc-lmb.cam.ac.uk/harry/pre/aimless.html
Phaser, McCoy et al.
Coot, Emsley et al., https://www2.mrc-lmb.cam.ac.uk/personal/pemsley/coot/
Phenix.refine, Afonine et al., https://phenix-online.org/download/
Modeller 9.16, Fiser et al., https://salilab.org/modeller/
Autodock4, Morris et al., https://autodock.scripps.edu/download-autodock4/
MacroModel Release 2019-3, Schrödinger Maestro Harder et al., https://newsite.schrodinger.com/platform/products/macromodel/
Zen, ZEISS, https://www.zeiss.com/microscopy/de/produkte/software/zeiss-zen.html
Sigma Plot, Systat Software GmbH, https://systatsoftware.com/sigmaplot
FlowJo™ Software, BD Life Sciences, https://www.bdbiosciences.com/en-de/products/software/flowjo-v10-software
Prism 8, GraphPad Software, https://www.graphpad.com
Origin 7/ Origin 8, Origin Lab, https://www.originlab.com
DiaTrack 3.0 software, Vallotton et al.

For manuscripts utilizing custom algorithms or software that are central to the research but not yet described in published literature, software must be made available to editors and reviewers. We strongly encourage code deposition in a community repository (e.g. GitHub). See the Nature Portfolio guidelines for submitting code & software for further information.

Data

Policy information about availability of data

All manuscripts must include a data availability statement. This statement should provide the following information, where applicable:

- Accession codes, unique identifiers, or web links for publicly available datasets
- A description of any restrictions on data availability
- For clinical datasets or third party data, please ensure that the statement adheres to our policy

Source data for each figure are provided as individual files. Uncropped versions of all Western Blots are found in the Supplementary Information file in the section "Western Blots". The PBD file of the crystal structure is available here: https://www.rcsb.org/structure/6Z2S. Any further information related to the study will be available upon request by the lead contact, Georgios Tsiavaliaris (Tsiavaliaris.Georgios@mh-hannover.de). All compounds and plasmids generated in this study are available upon request.

Research involving human participants, their data, or biological material

Policy information about studies with human participants or human data. See also policy information about sex, gender (identity/presentation), and sexual orientation and race, ethnicity and racism.

Reporting on sex and gender

The coloinoid donor is male.

Reporting on race, ethnicity, or other socially relevant groupings

The coloinoid donor is of Caucasian descent.

Population characteristics

Healthy |Age: 35 year | Biopsy section: Transverse Colon (citation: table S2 in https://www.mdpi.com/1422-0067/24/18/14214)

Recruitment

The donor was a volunteer.

Ethics oversight

Human biopsy used for establishing organoid cultures was collected from transverse colon of a volunteered donor after informed consent and institutional review board of Hannover Medical School (MHH) with approval number 8536_BO_K_2019 with the title "Deciphering the transport metabolomes of SLC26A3 and SLC26A6 during alkalization of different segments of the human gastrointestinal tract" from 26 June 2019.

Note that full information on the approval of the study protocol must also be provided in the manuscript.

Field-specific reporting

Please select the one below that is the best fit for your research. If you are not sure, read the appropriate sections before making your selection.

[x] Life sciences   [ ] Behavioural & social sciences   [ ] Ecological, evolutionary & environmental sciences

For a reference copy of the document with all sections, see nature.com/documents/nr-reporting-summary-flat.pdf

Life sciences study design

All studies must disclose on these points even when the disclosure is negative.

Sample size	Sample size was random depending on the experiment.
Data exclusions	No data were excluded.
Replication	All experiments were successfully reproduced.
Randomization	Samples/organisms and material from donors were randomly allocated to treatment and control groups
Blinding	Investigators were aware of the colonoid donor information. Drug screening, data acquisition, animal sampling was blind. All data were collected automatically and not by human evaluation.

Reporting for specific materials, systems and methods

We require information from authors about some types of materials, experimental systems and methods used in many studies. Here, indicate whether each material, system or method listed is relevant to your study. If you are not sure if a list item applies to your research, read the appropriate section before selecting a response.

Materials & experimental systems

n/a	Involved in the study	
☐	☒	Antibodies
☐	☒	Eukaryotic cell lines
☒	☐	Palaeontology and archaeology
☐	☒	Animals and other organisms
☒	☐	Clinical data
☒	☐	Dual use research of concern
☒	☐	Plants

Methods

n/a	Involved in the study	
☒	☐	ChIP-seq
☐	☒	Flow cytometry
☒	☐	MRI-based neuroimaging

Antibodies

Antibodies used

anti-Myo9a, Thermo Fisher Scientific, Cat# PA5-59055; RRID:AB_2644379
anti-Myo9b, Proteintech, Cat# 12432-1-AP; RRID:AB_2148635
anti-paxillin, BD Biosciences, Cat# 610051; RRID:AB_397463
anti-ZO1, Thermo Fisher Scientific, Cat# 40-2200; RRID:AB_2533456
anti-pMLC2, Cell signalling, Cat# 3671; RRID:AB_330248
anti-NMIIA, Biolegend, Cat# 909801; RRID:AB_2565100
anti-NMIIB, Biolegend, Cat# 909901; RRID:AB_2749903
anti-pan Actin, Abcam, Cat# ab119952
anti- phospho-MYPT1 (Thr850), Sigma-Aldrich; Cat# 36-003; RRID:AB_310812
anti-vinculin, Sigma-Aldrich; Cat# V9131; RRID:AB_477629
anti-VASP from Jan Faix, Damiano-Guercio et al., 2020
anti-GAPDH, Sigma-Aldrich, Cat# CB1001-500UG
anti-BrDU, Invitrogen, Cat# MA3-071; RRID:AB_10986341
anti-RhoA, Santa Cruz Biotechnology Cat# 26C4
anti-24B10, Developmental Studies Hybridoma Bank 24B10; RRID: AB_528161
anti-CoraC, Developmental Studies Hybridoma Bank C615.16; RRID: AB_1161644
anti-FasII, Developmental Studies Hybridoma Bank 1D4 anti-Fasciclin II, RRID: AB_528235
anti-DE-Cadherin, Developmental Studies Hybridoma Bank DCAD2, RRID: AB_528120
anti-NrxIV, from Christian Klämbt
Rabbit IgG (H&L) Secondary Antibody Peroxidase Conjugated Pre-adsorbed, Rockland Cat# 611-103-122
AlexaFluor555-conjugated goat anti-rabbit, Thermo Fisher Scientific, Cat# A-21428; RRID:AB_2535849
AlexaFluor488-conjugated goat anti-mouse, Thermo Fisher Scientific, Cat# A-11029; RRID:AB_2534088
AlexaFluor488-conjugated goat anti-rat, Thermo Fisher Scientific, Cat# A-11006; RRID:AB_2534074
AlexaFluor488-conjugated goat anti-rabbit, Thermo Fisher Scientific, Cat# A-11008; RRID:AB_143165
IgG1 Cy3 goat anti-mouse, Jackson ImmunoResearch Laboratory, Cat# 115-165-205
AlexaFluor633-conjugated phalloidin, Thermo Fisher Scientific, Cat# A12379; RRID:AB_2534069
Atto633- conjugated phalloidin, ATTO-TECH, Cat# AD 633-8
iFlour™488 anti-rabbit, ATT Bioquest, Cat# 16608
Stabilized peroxidase conjugated goat anti-rabbit, HRP conjugated, Invitrogen, Cat# 32460; RRID: AB_1185567
Stabilized peroxidase conjugated goat anti-mouse, HRP conjugated, Invitrogen, Cat# 32430; RRID: AB_1185566

Validation	All antibodies were validated by the manufacturer and/or by the investigators; anti-VASP was validated in: Damiano-Guercio, J. et al., 2020; anti-NrxIV which was validated in: Stork, T. et al., 2009.

Eukaryotic cell lines

Policy information about cell lines and Sex and Gender in Research

Cell line source(s)	A549, ATCC CCL-185; RRID: CVCL_0023<br>B16-F1, ATCC CRL-6323; RRID:CVCL_0158<br>MLE-12, ATCC CRL-2110; RRID:CVCL_3751<br>CaCo-2, ATCC HTB-37; RRID:CVCL_0025<br>Calu-3, ATCC HTB-55; RRID:CVCL_0609<br>NIH/3T3, ATCC CRL-1658; RRID:CVCL_0594<br>HeLa, ATCC CRM-CCL-2 RRID:CVCL_0030<br>Primary macrophages, BALB/cJRj
Authentication	Cell line authentication was not preformed. All cell lines were derived from original vials.
Mycoplasma contamination	All cell lines were regularly tested for mycoplasma and were mycoplasma free.
Commonly misidentified lines (See ICLAC register)	We did not use commonly misidentified cell lines.

Animals and other research organisms

Policy information about studies involving animals; ARRIVE guidelines recommended for reporting animal research, and Sex and Gender in Research

Laboratory animals	Drosophila Melanogaster: Oregon R, wild-type strain Bloomington Drosophila Stock Center BDSC: 5 FBgn0003996<br>Mus musculus: BALB/cJRj, The Jackson Laboratory JAX:000651; RRID:IMSR
Wild animals	No wild animals were used.
Reporting on sex	Both male and female flies were studied to avoid limitations of the data generalizability.<br>Twenty four mice were randomly allocated into four experimental groups each consisting of six mice (3 males, 3 females).
Field-collected samples	No field collected studies.
Ethics oversight	Animal housing of BALB/cJRj mice and experimental procedures were approved by the animal welfare committee of the Hannover Medical School, complied with the German animal welfare legislation and were finally approved by the Lower Saxony State Office for Consumer Protection and Food Safety (LAVES, AZ 33.12-42502-04-22-00021).

Note that full information on the approval of the study protocol must also be provided in the manuscript.

Plants

Seed stocks	-
Novel plant genotypes	-
Authentication	-

Flow Cytometry

Plots

Confirm that:

- [ ] The axis labels state the marker and fluorochrome used (e.g. CD4-FITC).
- [x] The axis scales are clearly visible. Include numbers along axes only for bottom left plot of group (a 'group' is an analysis of identical markers).
- [x] All plots are contour plots with outliers or pseudocolor plots.
- [x] A numerical value for number of cells or percentage (with statistics) is provided.

Methodology

Sample preparation	Flow cytometry was only applied in fixed cells in this study for quantification purposes.<br>For FACS experiments, cells were fixed and stained in solution with Atto488 phalloidin (0.5 µM, Atto-tec) to quantify F-actin and DAPI (1:500, 1mg/ml, Sigma) to perform cell cycle analysis as described in the section Immunofluorescence. Analysis was performed FlowJo™ v10.8 Software (BD Life Sciences).
Instrument	BD FACSAria III Fusion
Software	For cell cycle analysis (univariate modeling) we used the CellCycle module of FlowJo (Version 10.9.0), using the Watson pragmatic algorithm (Cytometry 8:1-8, 1987).
Cell population abundance	10.000 total events were analyzed per sample.
Gating strategy	Gating of cells based on scatter properties (FSC/SSC), exclusion of doublets (SSC-A/SSC-W), gating on phalloidin positive cells (phalloidin Atto488).

- [x] Tick this box to confirm that a figure exemplifying the gating strategy is provided in the Supplementary Information.
